# Mapping awareness of breast and cervical cancer risk factors, symptoms and lay beliefs in Uganda and South Africa

**DOI:** 10.1371/journal.pone.0240788

**Published:** 2020-10-22

**Authors:** J. Moodley, D. Constant, A. D. Mwaka, S. E. Scott, F. M. Walter

**Affiliations:** 1 Women’s Health Research Unit, School of Public Health and Family Medicine, Faculty of Health Sciences, University of Cape Town, Observatory, Cape Town, South Africa; 2 Cancer Research Initiative, Faculty of Health Sciences, University of Cape Town, Observatory, Cape Town, South Africa; 3 South African Medical Research Council Gynaecology Cancer Research Centre, Faculty of Health Sciences, University of Cape Town, Observatory, Cape Town, South Africa; 4 Department of Medicine, School of Medicine, College of Health Sciences, Makerere University, Kampala, Uganda; 5 Centre for Oral, Clinical and Translational Sciences, Faculty of Dentistry, Oral and Craniofacial Sciences, King’s College London, London, United Kingdom; 6 The Primary Care Unit, Department of Public Health & Primary Care, University of Cambridge, Cambridge, United Kingdom; University of the Witwatersrand, SOUTH AFRICA

## Abstract

**Background:**

Breast and cervical cancer are leading causes of cancer burden in Sub-Saharan Africa (SSA). We measured breast and cervical cancer symptom and risk factor awareness and lay beliefs in Uganda and South Africa (SA).

**Methods:**

Between August and December 2018 we conducted a cross-sectional survey of women ≥18 years in one urban and one rural site per country. Households were selected using systematic random sampling, then one woman per household randomly selected to participate. Data were collected by interviewers using electronic tablets customised with the locally validated African Women Awareness of Cancer (AWACAN) tool. This has unprompted questions (testing recall) followed by prompted questions (testing recognition) on risk factor, symptom awareness and lay beliefs for breast and cervical cancer. Mann Whitney and Kruskal Wallis tests were used to compare the association between socio-demographic variables and outcomes. Poisson regression with robust variance was conducted to identify independent socio-demographic predictors.

**Results:**

Of the 1758 women interviewed, 90.8% had heard of breast and 89.4% of cervical cancer. 8.7% recalled at least one breast risk factor and 38.1% recalled at least one cervical cancer risk factor. 78.0% and 57.7% recalled at least one breast/cervical cancer symptom respectively. Recognition of risk factors and symptoms was higher than recall. Many women were unaware that HPV, HIV, and not being screened were cervical cancer risk factors (23.7%, 46.8%, 26.5% respectively). In SA, urban compared to rural women had significantly higher symptom and risk factor awareness for both cancers. In Uganda married women/living with a partner had higher awareness of breast cancer risk factors and cervical cancer symptoms compared to women not living with a partner. Women mentioned several lay beliefs (e.g. putting money in their bra as a breast cancer risk factor).

**Conclusion:**

We identified gaps in breast and cervical cancer symptom and risk factor awareness. Our results provide direction for locally targeted cancer awareness intervention programs and serve as a baseline measure against which to evaluate interventions in SSA.

## Introduction

Breast and cervical cancer are the leading causes of cancer morbidity and mortality among women in Sub-Saharan Africa (SSA) [1]. Cervical cancer remains the leading cause of female cancer deaths in SSA with mortality rates as high as 40 per 100 000 in Uganda [2]. Breast cancer is the most common cancer in SSA and although incidence rates are lower than those of high-income countries, mortality rates in some SSA countries are similar [2]. For example, age-standardised incidence rates for breast cancer in the United Kingdom (UK) and South Africa (SA) are 94 and 49 per 100 000 women respectively, whilst age-standardised mortality rates in the UK and SA are 14 and 16 per 100 000 women respectively [2]. Most SSA countries do not have cervical or breast cancer screening programs and the majority of cancers are diagnosed symptomatically and at an advanced stage [3]. Late-stage at presentation is a major contributor to low breast and cervical cancer survival rates [4, 5].

Early stage presentation is a key goal of comprehensive cancer policies as it enables more opportunities for curative treatment and improved prognosis among those treated [3]. Prompt help-seeking in the presence of symptoms may lead to earlier-stage diagnoses, and may be influenced by factors such as symptom and risk factor awareness [6–10]. Individual and community understanding of cancer risk and symptoms can include lay beliefs, which may also influence risk reduction and help-seeking behaviours [9, 11].

Understanding the nature and predictors of cancer symptom and risk factor awareness is vital to underpin the development of locally relevant interventions. Programs aimed at raising public and lay health worker awareness of breast and cervical cancer have shown promise in promoting help-seeking behaviour and down-staging disease [6, 12]. Studies have suggested differences in levels of cancer knowledge between African countries. Furthermore, there may also be differences between urban and rural settings within a country [8, 13], underscoring the need for geographically targeted intervention programs.

Most studies measuring breast and cervical cancer awareness in Africa have been conducted in hospital settings among women already diagnosed with the disease [8, 9, 14]. Fewer studies report awareness at a community level among undiagnosed women where levels of cancer symptom and risk factor awareness and beliefs may differ. Furthermore, most studies in SSA, whether hospital or community-based have not used locally validated measures [15]. The aim of this study was to measure breast and cervical cancer symptom and risk factor awareness and lay beliefs in urban and rural settings in Uganda and SA using a culturally relevant, locally validated measurement tool.

## Materials and methods

### Study design

We conducted a community-based cross-sectional survey of women aged 18 years and older in one urban and one rural site in Uganda and SA (total of 4 sites). Uganda is a low-income country with a population of 34.9 million [16]. Both the urban and rural study sites were in Northern Uganda, the poorest of the four regions in Uganda. SA is a middle-income country with a population of 58.8 million [17]. Our urban SA study site was in the Western Cape Province, one of the wealthier of the nine provinces in the country, whereas our rural site was in the Eastern Cape Province which has very low levels of wealth [18].

### Public involvement

The African Women Awareness of Cancer (AWACAN) project was set-up to improve timely cancer diagnosis [19]. At the start of the AWACAN project, community-based project advisory committees (PACs) were set up in each county. The PACs included local government councillors, traditional leaders as well as representatives from the cancer registry and public sector health services (health service providers and managers). The AWACAN breast and cervical cancer tool used in the survey was developed with inputs from PAC members. In this study, PAC members assisted in gaining access to communities, recruiting local women as field workers in each site and provided guidance on field worker safety. AWACAN results have been discussed with PAC members and field workers in a series of meetings.

### Participants

At each site, households were selected using systematic random sampling. The name of each woman in the household was written on a separate piece of paper, folded and placed in a “hat”. Random selection of a participant was then done by picking one from the set of folded papers. If the woman selected did not want to participate or there were no women in the household, fieldworkers moved to the next marked household, noting the reason for non-participation. If the selected woman from the household was away, fieldworkers returned for an interview later. Women unable to speak either Acholi, isiXhosa or English; women with a history of breast or cervical cancer and; woman younger than 18 years were excluded.

### Data collection

Data were collected by trained interviewers using a hand-held electronic tablet customised with the AWACAN breast and cervical cancer tool. Details of the content, development and validation of the tool, are described elsewhere [15]. Women were interviewed face-to-face in a private space in their homes, either in their local language (isiXhosa or Acholi) or in English depending on their preference.

#### Measures of socio-demographics

We collected the following socio-demographic details: age, relationship status, highest educational level, work status i.e. whether doing paid work, and information on access to assets. Asset variables in the questionnaire were based on previous research in SA using General Household Surveys [20]. We constructed a composite asset index to describe socioeconomic status using principal component analysis (PCA) on a model that included asset variables with a positive correlation at p≤0.01. Only five variables satisfied this condition (access to piped water, electricity, radio, television and the internet). To ensure consistency between countries, we used the same set of variables for both SA and Uganda, treating each country individually. The asset index was then divided into terciles, as it did not sufficiently discriminate between households to allow for more than three levels. To check the validity of the asset index terciles, we tested for association with other key variables, namely paid work and highest education level; all associations were significant at p<0.001.

#### Measures of cancer awareness

We measured cancer awareness by asking whether the participant had heard of breast/cervical cancer and whether they knew of any family members, friends or neighbours who had breast/cervical cancer. Risk factor and symptom awareness was assessed using an open/unprompted question followed by closed/prompted question format as laid out in the AWACAN breast and cervical cancer tool [15]. As risk factor questions related specifically to either breast or cervical cancer, these questions were posed only to participants that had heard of breast or cervical cancer. We asked all participants about symptom awareness as these questions as these were framed around whether a symptom was viewed as a sign of something serious. Breast cancer risk factor open questions (measuring recall) read “Please can you name as many things as you can think of that could increase any woman’s chances of getting breast cancer”. Open questions for symptoms read “Name as many symptoms or signs of breast cancer as you can think of”. Closed questions (measuring recognition) asked “Could any of the following increase any woman’s chances of getting breast cancer” (13 items) and “Do you think the following could be signs of something serious or that something is wrong, such as breast cancer” (15 items) [15]. We used the AWACAN tool open and closed question format to measure recall and recognition of cervical cancer risk factors and symptoms. There were 11 closed questions assessing cervical cancer risk factor recognition and 11 questions assessing symptom recognition [15]. The AWACAN questionnaire includes prompted lay belief items: 6 breast cancer risk factors, 1 breast cancer symptom, 4 cervical cancer risk factors and 1 cervical cancer symptom [15]. Additional lay beliefs were identified during unprompted questioning.

For open questions, women scored ‘1’ if they mentioned a risk factor or symptom that corresponded to the list of closed questions, and ‘0’ if this was not mentioned. Scoring of open responses was done by two investigators (DC and JM). For closed questions measuring recognition women scored ‘1’ for ‘Yes’ and ‘0’ for ‘No’ or ‘Don’t know’. Scores for each response to the closed questions were summed to form separate knowledge scores for breast cancer risk factors (0–13), breast cancer symptoms (0–15), cervical cancer risk factors (0–11) and symptoms (0–11). Higher scores indicated better recognition of symptoms and risk factors and greater endorsement of lay beliefs. The median and the interquartile range (IQR) are reported for symptom and risk factor scores. Similar to other studies we dichotomised scores for risk factor and symptom awareness using the median as a cut-off for low and high awareness [21–23]. The median was classified as low awareness to achieve an even distribution between groups.

### Ethics

Ethics approval for the study was obtained from University of Cape Town, Faculty of Health Sciences Human Research Ethics Committee (HREC 544/2016), the Lacor Hospital Institutional Research Ethics Committee (LHIREC 027/11/2016) and the Ugandan National Council of Science and Technology (HS60ES). Consent forms were translated and administered in the participants’ preferred language. Written informed consent was obtained from all participants.

### Sample size and analysis

To detect differences in awareness scores of at least 10% between groups for the various comparisons (e.g. between countries, by urban/rural location, by age groups) assuming a two-sided two-sample test with alpha = 0.05, a standard deviation of 0.2 and allowing for a 15% non-response rate, we aimed to recruit 400 women at each site.

We analyzed the data using Stata version 15, using valid percentages to report socio-demographic data. Medians and interquartile ranges (IQR) were calculated for continuous variables. As Ugandan and SA socio-demographic indicators showed significant differences, we did the bivariate and multivariable analysis by country to best demonstrate these context specific effects. As none of the scores were normally distributed, we compared scores according to socio-demographic factors in bivariate analyses using Mann Whitney and Kruskal Wallis tests as appropriate, using a significance level of p ≤ 0.01. Poisson regression with robust variance was conducted to identify independent socio-demographic predictors for risk factor and symptom awareness. Prevalence ratios (PR) and 95% confidence intervals are reported. The regression models included the following variables: location, age, relationship status, highest education level, paid work and asset index.

## Results

Between August and December 2018, a total of 1941 households were visited in Uganda and SA, and 1758 participants recruited. The overall refusal rate was 6.3% and was highest in the urban Ugandan site (13.7%). S1 Appendix Participant recruitment provides details of site recruitment, reasons for refusal and ineligibility, and final enrolment numbers.

The results are presented in 3 sections: Section 1 outlines the Participant profile. The results on awareness of risk factors and symptoms as well as lay beliefs are presented under Section 2: Breast cancer and Section 3: Cervical cancer

### Section 1: Participant profile

The median age of respondents was 34 years (IQR 26–47). Participant characteristics by country and site are outlined in Table 1. A higher proportion of women in rural Uganda were married and had no or incomplete primary schooling compared to other sites. Overall, 66.3% of participants did no paid work, with the highest rate in rural SA (91.8%).

**Table 1:** Participant profile.

	South Africa		Uganda		Total
	Urban	Rural	Urban	Rural	
	n (%) n = 445	n (%) n = 428	n (%) n = 458	n (%) n = 427	n (%) n = 1758
**Age (years)**					
18–29	130 (30.4)	106 (25.2)	216 (47.2)	186 (44.0)	638 (36.9)
30–49	221 (51.6)	170 (40.4)	174 (38.0)	146 (34.5)	711 (41.1)
≥ 50	77 (18.0)	145 (34.4)	68 (14.9)	91 (21.5)	381 (22.0)
Median (IQR)	36 (28–45)	40 (29–54)	30 (24–40)	32 (23–46)	34 (26–47)
**Relationship status**					
Married/Living with a partner	185 (41.8)	200 (46.7)	300 (65.5)	314 (73.5)	999 (56.9)
No partner/not living with partner	224 (50.6)	174 (40.7)	36 (7.9)	17 (4.0)	451 (25.7)
Separated/Divorced/Widowed	34 (7.7)	54 (12.6)	122 (26.6)	96 (22.5)	306 (17.4)
**Highest educational level**					
No schooling & primary incomplete	22 (5.0)	162 (38.0)	171 (37.3)	328 (76.8)	683 (39.0)
Primary complete & secondary incomplete	196 (44.3)	178 (41.8)	167 (36.5)	84 (19.7)	625 (35.6)
Secondary complete or more	224 (50.7)	86 (20.2)	120 (26.2)	15 (3.5)	445 (25.4)
**Paid work**					
Yes	226 (51.6)	35 (8.2)	217 (47.6)	110 (25.8)	588 (33.7)
No	212 (48.4)	391 (91.8)	239 (52.4)	317 (74.2)	1159 (66.3)
**Asset index**					
Upper tercile	326 (73.3)	6 (1.4)	253 (55.2)	70 (16.4)	655 (37.3)
Middle tercile	79 (17.8)	182 (42.5)	122 (26.6)	210 (49.2)	593 (33.7)
Lower tercile	40 (9.0)	240 (56.1)	83 (18.1)	147 (34.4)	510 (29.0)
**Ever heard of breast/cervical cancer**					
Heard of breast cancer	406 (91.2)	342 (79.9)	445 (94.4)	403 (94.3)	1596 (90.8)
Heard of cervical cancer	366 (82.3)	342 (79.9)	452 (98.7)	411 (96.3)	1571 (89.4)

There were missing data for

Age: South Africa Urban; n = 17, Rural; n = 7. Uganda Rural; n = 4. Total; n = 28.

Relationship status: South Africa Urban; n = 2. Total; n = 2.

Highest educational level: South Africa Urban; n = 3, Rural; n = 2. Total; n = 5.

Paid work: South Africa Urban; n = 7, Rural; n = 2. Uganda Urban = 2. Total; n = 11.

Asset distribution differed by site. Women in urban SA had the most assets (73.3% at upper tercile), whilst those in rural SA had the fewest assets (56.1% at lower tercile). S2 Appendix provides details on participants’ assets by site and by country. Overall, two-thirds of women had access to a radio, half had access to television and just under half had access to the internet, with differences between countries and urban/rural sites. Most women (90.8%%) had heard of breast cancer; of those, 30.3% knew of someone with the disease. Most women (89.4%) had heard of cervical cancer, and of those, almost a third (29.0%) knew of someone with the disease.

### Section 2: Breast cancer

#### Breast cancer risk factor awareness

**Fig 1:**
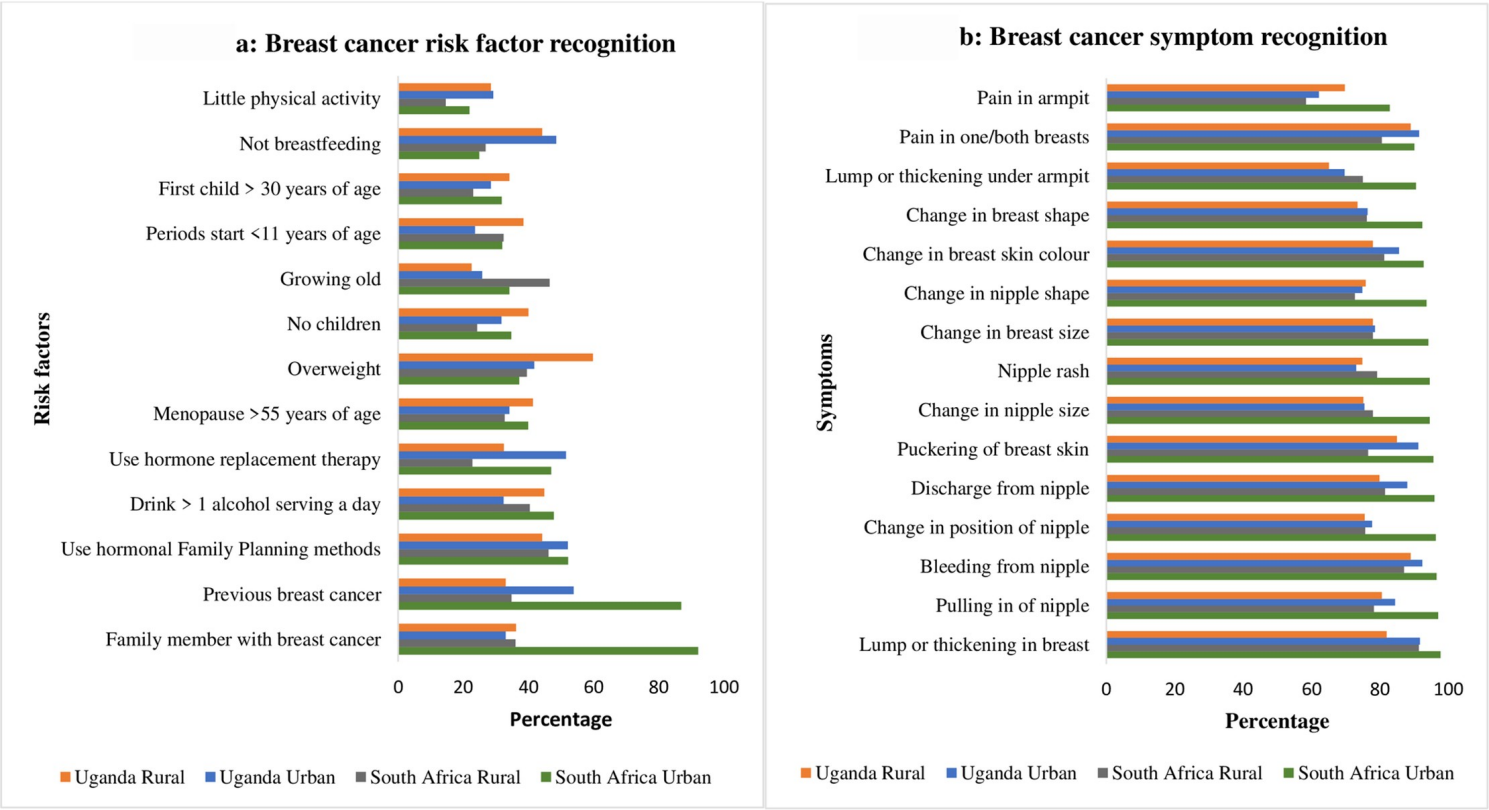
Breast cancer risk factor and symptom recognition by site.

Risk factor recall was low in all sites with only 8.7% of participants among those that had heard of breast cancer (n = 1596) able to recall at least one risk factor (S3 Appendix). Recognition of risk factors was better than recall with 95.2% of women able to recognize at least 1 of 13 risk factors. However, most women could only recognize less than half of the 13 risk factors (median risk recognition score = 4, IQR 3–7). Recall and recognition of individual risk factor and symptoms by site are provided in S3 Appendix. Overall, the most commonly recognized risk factor was a past history of breast cancer (52.9%.) and the least commonly recognized was doing little physical activity (24.1%). Fig 1A depicts the proportion of women recognizing each risk factor by site.

In SA, women living in an urban location, those aged between 30 and 49, those with paid work, and women with the highest asset index assets had greater awareness of breast cancer risk factors (Table 2, bivariate analysis). On regression urban location remained a significant predictor of greater awareness of breast cancer risk factors (PR 1.35, 95% CI 1.07–1.72). Women aged between 30 and 49 years also had significantly higher risk factor awareness compared to women younger than 30 years (PR 1.28; 95% CI 1.03–1.58) (S4 Appendix). In Uganda, relationship status, highest educational level and asset index were associated with risk factor recognition on bivariate analysis (Table 3). On multivariable analyses the only factor that remained significant was relationship status, women not living with a partner/having no partner had significantly lower risk awareness compared to women that were married/living with a partner (PR 0.37; 95% CI 0.19–0.73) (S5 Appendix)

**Table 2:** Socio-demographic predictors of breast risk factor and symptom awareness in South Africa.

	Recognized breast cancer risk factors N = 748*		Recognized breast cancer symptoms N = 873**	
	Median (Interquartile Range)	Test statistic, p-value	Median (Interquartile Range)	Test statistic, p-value
**Location**		z = 5.883, p<0.001		z = 12.839, p<0.001
Urban	5 (3–9)		15 (14–15)	
Rural	4 (2–6)		13 (10–14)	
**Age**		chi^2^ = 12.637, p = 0.002		chi^2^ = 3.760, p = 0.153
18–29	4 (2–7)		14 (12–15)	
30–49	5 (3–8)		14 (12–15)	
≥ 50	4 (3–6)		14 (11–15)	
**Relationship status**		chi^2^ = 3.128, p = 0.209		chi^2^ = 4.500, p = 0.105
Married/Living with a partner	4 (3–7)		14 (12–15)	
No partner/not living with partner	4 (2–7)		14 (12–15)	
Separated/Divorced/Widowed	4 (2–6)		14 (10–15)	
**Highest educational level**		chi^2^ = 4.427, p = 0.109		chi^2^ = 42.051, p<0.001
No schooling to primary incomplete	4 (2–7)		13 (10–14)	
Primary complete to secondary incomplete	4 (2–7)		14 (12–15)	
Secondary complete or more	4 (3–8)		15 (13–15)	
**Paid work**		z = -5.302, p<0.001		z = -5.691, p<0.001
Yes	5 (3–9)		15 (13–15)	
No	4 (2–6)		14 (11–15)	
**Asset index**		chi^2^ = 12.158, p = 0.002		chi^2^ = 66.029, p<0.001
Upper Tercile	5 (3–8)		15 (13–15)	
Middle Tercile	4 (2–7)		14 (11–15)	
Lower Tercile	4 (2–7)		13 (10.5–15)	

*N = [number enrolled–number that had not heard of breast cancer]

** N = number enrolled.

Where 2 categories of independent variables are compared: z statistic is reported for Mann-Whitney two sample test.

Where 3 categories of independent variables are compared: chi-squared statistic is report for Kruskal-Wallis rank test.

**Table 3:** Socio-demographic predictors of breast risk factor and symptom awareness in Uganda.

	Recognized breast cancer risk factors N = 848*		Recognized breast cancer symptoms N = 885**	
	Median (Interquartile Range)	Test statistic, p-value	Median (Interquartile Range)	Test statistic, p-value
**Location**		z = 0.882, p = 0.378		z = -1.171, p = 0.242
Urban	5 (2–7)		13 (11–15)	
Rural	5 (3–7)		13 (11–15)	
**Age**		chi^2^ = 6,865, p = 0.032		chi^2^ =, 7.813 p = 0.020
18–29	4 (2–7)		13 (10–14)	
30–49	5 (3–8)		13 (11–15)	
≥ 50	5 (3–7)		13 (9–15)	
**Relationship status**		chi^2^ = 17.698, p<0.001		chi^2^ = 8.291, p = 0.016
Married/Living with a partner	5 (3–7)		13 (11–15)	
No partner/not living with partner	2 (1–5)		12 (10–14)	
Separated/Divorced/Widowed	5 (3–7)		13 (9–14)	
**Highest educational level**		chi^2^ = 13.686, p = 0.001		chi^2^ =, 6.817 p = 0.033
No schooling to primary incomplete	5 (3–7)		13 (10–15)	
Primary complete to secondary incomplete	4 (2–7)		13 (10–14)	
Secondary complete or more	4 (2–6)		13 (12–15)	
**Paid work**		z = 2.114, p = 0.035		z = 0.057, p = 0.954
Yes	4 (2–7)		13 (10–15)	
No	5 (3–7)		13 (10–15)	
**Asset index**		chi^2^ = 9.257, p = 0.010		chi^2^ = 4.203, p = 0.122
Upper Tercile	4 (2–7)		13 (11–15)	
Middle Tercile	5 (3–7)		13 (10–15)	
Lower Tercile	5 (3–7)		13 (10–14)	

*N = [number enrolled–number that had not heard of breast cancer]

** N = number enrolled.

Where 2 categories of independent variables are compared: z statistic is reported for Mann-Whitney two sample test.

Where 3 categories of independent variables are compared: chi-squared statistic is report for Kruskal-Wallis rank test.

#### Breast cancer symptom awareness

Breast cancer symptom recall and recognition was high (S3 Appendix Recalled and recognized breast cancer risk factors and symptoms by site). Overall, 78.0% of women could recall at least one breast cancer symptom, most commonly a breast lump or thickening. Breast cancer symptom recognition was higher than recall with 99.0% of participants recognizing at least one of 15 symptoms and an overall median symptom recognition score of 14 (IQR 11–15). A breast lump was the most recognized symptom (90.8% of all participants); Fig 1B shows the proportion of women recognizing each symptom by site.

In SA, location, highest education level, paid work and asset index) was associated with symptoms awareness (Table 2, bivariate analysis). On regression analysis women living in the urban site were significantly more likely to know about breast cancer symptoms compared to rural women (PR 2.83; 95% CI 2.24–3.57), as were women who had completed secondary education or more compared to those with no or primary incomplete educational levels (PR 1.41; 95% CI 1.04–1.91) (S4 Appendix). Women doing paid work were less aware of breast symptoms compared to those not doing paid work (PR 0.84; 95% CI 0.71–0.98) (S4 Appendix). For Uganda, none of the socio-demographic factors were associated with breast cancer symptom awareness on bivariate analysis (Table 3), but on multivariable analysis older age was significantly associated with greater symptom awareness (women aged between 30 and 49 years compared to women younger that 30 years old PR 1.37; 95% CI 1.15–1.63 and, women 50 years and older compared to women younger than 30 years old PR 1.32; 95% CI 1.04–1.69) (S5 Appendix).

#### Breast cancer lay beliefs

On unprompted questioning, some women (21.1%) mentioned putting money in their bra as a possible breast cancer risk factor. This lay belief was strongly endorsed in prompted questioning, particularly among women in urban SA (97.8%). Other lay beliefs relating to undergarments were also strongly endorsed in prompted questions (S6 Appendix Unprompted and prompted breast and cervical cancer lay beliefs). Lay beliefs related to being bewitched were not commonly held, mentioned by only 0.8% of all participants in unprompted questioning and endorsed by 22.2% in prompted questioning.

### Section 3: Cervical cancer

#### Cervical cancer risk factor awareness

Of the 1571 participants that had heard of cervical cancer, 38.1% were able to recall at least one risk factor. In Uganda, having multiple sexual partners was the most commonly recalled risk factor. In urban SA having unprotected sex was the most commonly recalled risk factor, whilst in rural SA having other sexually transmitted diseases was most commonly recalled. Not going for regular screening and having an HPV infection were very poorly recalled, with <1% of all participants mentioning either of these risk factors. S7 Appendix gives information on recall and recognition of each cervical cancer risk factor and symptom by site.

**Fig 2:**
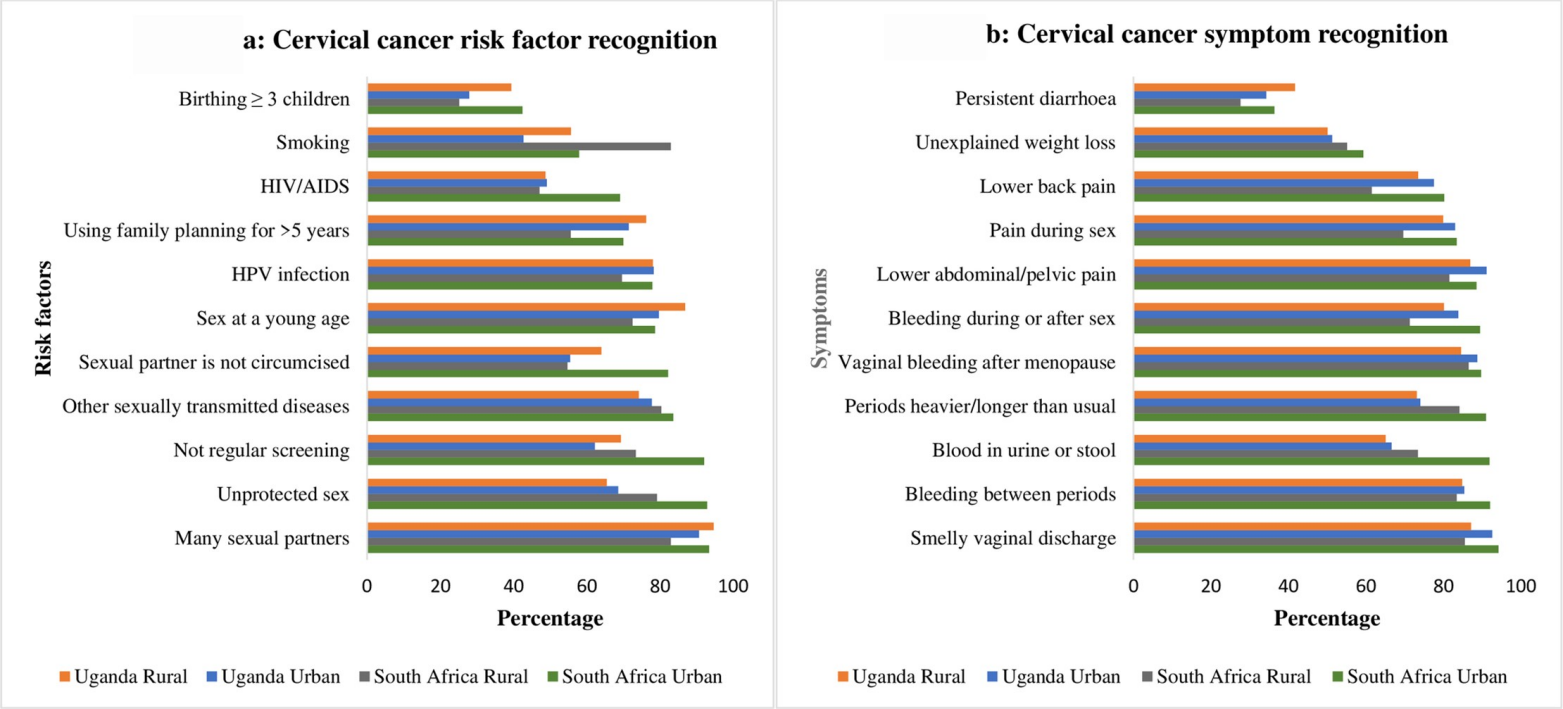
Cervical cancer risk factor and symptom recognition by site.

Recognition of risk factors was better than recall, with 99.1% recognizing at least 1 of 11 risk factors. Overall, the median score for risk factor recognition was 8 (IQR 6–9), and the most commonly risk factor recognized was having many sexual partners. Of the participants 21.8% in Uganda and 26.1% in SA, did not recognize that getting an HPV infection was a risk factor for cervical cancer (Fig 2). Some women did not recognize that not going for regular screening was a risk factor for cervical cancer, with considerable variation across sites (7.9% in urban SA, 26.6% in rural SA, 37.8% in urban Uganda and 30.7% in rural Uganda).

For SA, geographical location and paid work were significantly associated with cervical cancer risk awareness on bivariate analysis (Table 4). Only living in an urban location remained significantly associated with higher cervical cancer risk factor awareness in regression analysis (PR 1.55; 95% CI 1.22–1.99) (S8 Appendix). For Uganda, location, highest educational level and asset index were associated with cervical cancer risk recognition on bivariate analysis (Table 5). Factors that remained significant on regression analysis were secondary or higher educational level compared to no schooling/incomplete primary education (PR 0.66; 95% 0.46–0.96); paid work (PR 0.82; 95% CI 0.67–0.99) (S9 Appendix).

**Table 4:** Socio-demographic predictors of cervical cancer risk factor and symptom awareness in South Africa.

	Recognized cervical cancer risk factors N = 708*		Recognized cervical cancer symptoms N = 873**	
	Median (Interquartile Range)	Test statistic, p-value	Median (Interquartile Range)	Test statistic, p-value
**Location**		z = 5.717, p<0.001		z = 6.916, p = <0.001
Urban	9 (7–10)		9 (8–11)	
Rural	8 (6–9)		8 (6–10)	
**Age**		chi^2^ = 1.675 p = 0.433		chi^2^ = 6.050, p = 0.049
18–29	8 (6–10)		9 (7–10)	
30–49	8 (7–10)		9 (8–10)	
≥ 50	8 (7–9)		9 (7–10)	
**Relationship status**		chi^2^ = 3.033, p = 0.220		chi^2^ = 2.194, p = 0.334
Married/Living with a partner	8 (7–10)		9 (7–10)	
No partner/not living with partner	8 (6–10)		9 (7–10)	
Separated/Divorced/Widowed	7.5 (6–9)		9 (6.5–10)	
**Highest educational level**		chi^2^ = 3.289, p = 0.193		chi^2^ = 2.753, p = 0.252
No schooling to primary incomplete	8 (6–10)		9 (7–10.5)	
Primary complete to secondary incomplete	8 (7–10)		9 (7–10)	
Secondary complete or more	8 (6–10)		9 (7–10)	
**Paid work**		z = -3.177, p = 0.002		z = -3.299 p = 0.001
Yes	9 (7–10)		9 (8–11)	
No	8 (6–9)		9 (7–10)	
**Asset index**		chi^2^ = 8.422, p = 0.015		chi^2^ = 8.954, p = 0.011
Upper Tercile	9 (7–10)		9 (8–10)	
Middle Tercile	8 (6–10)		9 (7–10)	
Lower Tercile	8 (6–10)		9 (7–10)	

*N = [number enrolled–number that had not heard of cervical cancer]

** N = number enrolled.

Where 2 categories of independent variables are compared: z statistic is reported for Mann-Whitney two sample test.

Where 3 categories of independent variables are compared: chi-squared statistic is report for Kruskal-Wallis rank test.

**Table 5:** Socio-demographic predictors of cervical cancer risk factor and symptom awareness in Uganda.

	Recognized cervical cancer risk factors N = 863*		Recognized cervical cancer symptoms N = 885**	
	Median (Interquartile Range)	Test statistic, p-value	Median (Interquartile Range)	Test statistic, p-value
**Location**		z = 3.179, p = 0.002		z = -0.129, p = 0.898
Urban	7 (5–9)		9 (7–10)	
Rural	8 (6–9)		9 (6–10)	
**Age**		chi^2^ = 1.875, p = 0.392		chi^2^ = 5.847 p = 0.054
18–29	8 (6–9)		9 (7–10)	
30–49	8 (6–9)		9 (7–10)	
≥ 50	7 (6–9)		9 (6–10)	
**Relationship status**		chi^2^ = 6.512, p = 0.039		chi^2^ = 8.956, p = 0.011
Married/Living with a partner	8 (6–9)		9 (7–10)	
No partner/not living with partner	6.5 (4–9)		8 (6–9)	
Separated/Divorced/Widowed	8 (5–9)		9 (6–10)	
**Highest educational level**		chi^2^ = 18.840, p<0.001		chi^2^ = 1.430, p = 0.489
No schooling to primary incomplete	8 (6–10)		9 (6–10)	
Primary complete to secondary incomplete	8 (6–9)		9 (7–10)	
Secondary complete or more	7 (5–8)		9 (7–10)	
**Paid work**		z = 2.351, p = 0.019		Z = 1.805, p = 0.071
Yes	7 (6–9)		9 (6–10)	
No	8 (6–9)		9 (7–10)	
**Asset index**		chi^2^ = 16.046, p<0.001		chi^2^ = 3.976, p = 0.137
Upper Tercile	7 (5–9)		9 (7–10)	
Middle Tercile	8 (6–10		9 (7–10.5)	
Lower Tercile	8 (5–10)		9 (6–10)	

*N = [number enrolled–number that had not heard of cervical cancer]

** N = number enrolled.

Where 2 categories of independent variables are compared: z statistic is reported for Mann-Whitney two sample test.

Where 3 categories of independent variables are compared: chi-squared statistic is report for Kruskal-Wallis rank test.

#### Cervical cancer symptom awareness

In total 57.7%, of women were able to recall at least one symptom of cervical cancer. Recognition of symptoms was higher than recall, with 97.9% of participants able to recognize at least one symptom. Overall, the most commonly recognized symptom was a smelly vaginal discharge (89.9%) and more than 80.6% of women recognized unusual vaginal bleeding as a symptom. The least commonly recognized symptom overall was persistent diarrhea (35.0%) (Fig 2B).

In SA, women in the urban site and those doing paid work had significantly higher cervical cancer symptom awareness on bivariate analysis (Table 4). After adjusting for all included variables, factors that were significantly associated with higher cervical cancer risk recognition in SA were living at an urban site (PR 1.79; 95% CI 1.41–2.27), older age (women aged 30–50 years, PR 1.37; 95% CI 1.10–1.71 and women ≥50 years and older PR 1.47; 95% CI 1.13–1.92, each compared to women younger than 30 years) and, women with the least compared to the most assets (PR 1.34; 95% CI 1.05–1.70) (S8 Appendix). In Uganda, women with no partner/not living with a partner had significantly lower symptom awareness compared to women that were married or living with a partner (PR 0.60; 95% CI 0.37–0.98) (S9 Appendix).

#### Cervical cancer lay beliefs

Overall, poor personal hygiene was most frequently raised as a risk factor for cervical cancer both on prompted (92.9%) and unprompted questioning (33.7%), although fewer women in rural SA raised or endorsed this belief (S6 Appendix). When prompted, most women also endorsed vaginal insertion of herbs/creams/object as a possible risk factor (85.4% of all women). As with breast cancer, lay beliefs related to being bewitched were not commonly held, mentioned by <0.5% of all participants in unprompted questioning and endorsed by 22.3% in prompted questioning.

## Discussion

This is the first study measuring community level breast and cervical cancer awareness across diverse settings in SSA using a locally validated questionnaire. We found that, although most women had heard of both cancers in urban and rural settings in SA and Uganda, knowledge of risk factors and symptoms was low, particularly when measured in an unprompted mode (testing recall). In line with findings from previous studies [10, 14, 24, 25], we found that risk factor and symptom awareness was higher with prompted questioning. With prompted questioning, participants were provided with cues (the questions) and limited response options, thus making the recognition task easier than the recall task of unprompted questioning. In addition the prompted mode of questioning is prone to guessing, which could in part account for higher scores.

Awareness of risk factors for breast cancer was very low in all four of our study sites, both on unprompted and prompted questioning. Similar to other studies in low-and middle-income African settings [8, 9, 14, 26], we found poor levels of knowledge for factors which are responsive to interventions such as physical exercise and being overweight. Information on these risk factors need to be included in cancer awareness programs. In addition, as these risk factors are common to other non-communicable diseases (NCDs) such as cardiovascular disease and diabetes, NCD behavioral intervention programs and service platforms could be used to educate women about the link between these factors and breast cancer [3]. Adapting existing interventions to include additional messaging could be an efficient and cost-effective approach to addressing low breast cancer risk factor awareness.

Most women were aware that a lump was a possible symptom of breast cancer, whereas armpit changes were not well recognized. Again, these findings have been reported from studies in similar settings [8, 14], and have also been associated with delayed presentation [7, 27, 28]. Differences in levels of awareness of different symptoms could be related to the emphasis placed by cancer education initiatives on a breast lump compared to other symptoms. As one in six women with breast cancer may present with a non-lump symptom [27], breast cancer awareness programs need to also highlight these less well-known symptoms in order to contribute to more timely diagnosis of breast cancer.

Cervical cancer has been recognized as a major public health problem in SA and Uganda, with both countries offering public sector prevention programs that include HPV vaccination (SA and Uganda) and cervical cancer screening (SA) [29, 30]. Of concern is the finding that in our study many women in rural SA and urban and rural Uganda did not identify lack of screening as a risk factor for cervical cancer. Poor screening awareness has also been reported elsewhere in Africa [31, 32]

The association between HIV and cervical cancer is well established [33, 34] and most screening programs offer more frequent screening for HIV infected women [29, 35] In SA cervical cancer screening guidelines have been incorporated into HIV services for HIV infected women [29]. Despite these efforts, almost half the women in our study did not recognize that HIV was a risk factor for cervical cancer. Efforts to improve community awareness around the benefits of screening and about the link between HIV and cervical cancer may help increase screening coverage and reduce the prevailing high cervical cancer incidence.

Despite both SA and Uganda having public sector HPV vaccination programs [29, 30] almost a quarter of women did not recognize that HPV infection was a risk factor for cervical cancer. Interestingly, in both SA and Uganda having many sexual partners was the most recognized cervical cancer risk factor, suggesting that although women know that sexual intercourse is linked to cervical cancer, the underlying mechanism (HPV infection) is not understood by most. Interventions to help women understand the link between this risk factor and cervical cancer might help increase HPV vaccination uptake. Encouragingly most women recognized abnormal vaginal bleeding and smelly vaginal discharge as symptoms of cervical cancer.

We found breast and cervical cancer symptom and cervical cancer risk factor awareness was lower in rural compared to urban sites in SA, but not in Uganda. The SA findings confirm those of a much smaller study conducted in SA in 2002 [36]. These differences may be related to the high levels of inequity in SA as indicated by the Gini co-efficient of 0.6 for SA compared to a co-efficient of 0,4 for Uganda [37]. Inequity between urban and rural areas is also likely to include differences in access to evidence-based cancer information, prevention messaging and access to screening services. This is seen in the 2016 South African Demographic and Health Survey which reported differences in women accessing Pap smear screens by province as well as by urban versus non-urban residence [18]. Our study highlights the importance of investigating cancer awareness and knowledge in both urban and rural settings when developing evidence to inform and implement cancer awareness interventions. Our study also provides information on women’s access to the radio, television and internet by geographic area which could inform how best to deliver awareness interventions in the different settings.

Similar to other studies we also found that in SA higher education levels were associated with greater breast cancer symptom awareness [9]. Our finding that women doing paid work in SA had lower breast cancer symptom awareness compared to those not doing paid work was surprising and further research is required to confirm this finding.

For Uganda, the socio-demographic variables that were significantly associated with breast cancer awareness were relationship status and age, with women not living with a partner/not having a partner and women younger that 30 years compared to older women reporting lower awareness of breast risk factors and symptoms respectively. A study on breast cancer awareness among women recently diagnosed with breast cancer in Uganda also showed that unmarried women had lower breast cancer awareness [8]. Similar to a study in Kinshasa we found that women not living with a partner/not having a partner in Uganda had lower cervical cancer awareness compared to women living with a partner [21]. These findings underscore the importance of cancer awareness programs reaching specific target groups.

Belief in witchcraft has been shown to be important in disease conceptualization for both communicable and non-communicable diseases, although less research has been undertaken on belief of witchcraft as cause of cancer [26, 38]. We found that few participants in Uganda and SA identified witchcraft as a risk factor for either cancer. This finding may be the result of changing beliefs, but needs to be interpreted with caution given that we only used one question to assess this belief. Witchcraft beliefs are complex and community-specific. Future studies would benefit from using multiple items to measure this complexity as well as qualitative research to better explore community- specific beliefs.

We identified important lay beliefs including: putting money in a bra and wearing a tight bra (the commonest lay beliefs held in SA and Uganda respectively about increasing risk for breast cancer); poor personal hygiene and insertion of objects, creams or herbs (the most commonly held lay beliefs about increasing risk for cervical cancer). Other African studies have reported on the belief that vaginal insertion of creams and objects, employed as a means to increase partner sexual satisfaction or as part of a hygiene practice, could be a cause of cervical cancer [39–41]. Beliefs related to wearing a tight bra have also been reported previously [9]. If individual behavior is directed by lay beliefs this could result in a false sense of protection against cancer or a misattribution of symptoms and delayed help-seeking. Therefore, it is important that intervention programs not only provide information on evidence-based risk factors and symptoms but also address lay beliefs.

A major strength of our study was the use of a locally validated questionnaire—this enabled comparison across our study sites in urban and rural locations in two countries, and will allow for accurate measurement of changes in awareness over time. Many studies measuring cancer awareness have been conducted at health facilities amongst women diagnosed with cancer, with information assumed to hold true for undiagnosed women. A strength of our study was that it was community-based with a random selection of homesteads across different locations, thereby minimizing selection bias. A further strength was that our questionnaire was available in local languages (Acholi in Uganda and isiXhosa in SA) as well as in English, allowing for true community participation—this together with the PAC support facilitated community access and could explain our very high response rate. The use of tablets customized with the questionnaires and with off-line capability further aided efficient data collection, particularly in the rural areas with poor internet connectivity.

A limitation of our study was that our questions assessing recognition of breast and cervical cancer symptoms were slightly different from the recall questions for breast and cervical cancer symptoms. For example for breast cancer we asked specifically “Please would you name as many symptoms or signs of breast cancer as you can think of?” whereas for recognition we expanded the question to “Can you tell me if you think the following could be signs of something serious or that something is wrong, such as breast cancer?” This was done to acknowledge that symptom interpretation as something “serious” is likely to drive help-seeking rather than just attribution specifically to cancer. But it does mean we are not comparing like with like with recall (cancer symptoms only) vs recognition. The differences in levels of knowledge measured by unprompted versus prompted questioning highlight the need for careful consideration of how knowledge is measured and compared between studies. A further limitation of our study was that the development of our asset index was informed by the few socio-demographic variables we collected. It is possible that an asset index informed by more variables might have elicited an association between cancer awareness and socio-economic status as has been described in other studies [10, 42]. Addressing the two leading female cancers in SSA in one study is a resourceful way to maximise data collection from women and minimises duplication between studies. However, this did result in a long questionnaire and a possible limitation of this approach is participant fatigue.

## Conclusion

Our study identified important and specific gaps in community breast and cervical cancer risk and symptom awareness as well as cancer-related lay beliefs in rural and urban settings in SA and Uganda. These results could inform targeted intervention programs in similar urban and rural locations in both countries e.g. media campaigns to increase awareness of the non-lump symptoms for breast cancer. Our findings also provide an important baseline measure for the evaluation of future interventions.

## Supporting information

S1 Appendix Participant recruitment. (DOCX)

S2 Appendix Participants’ assets by site, country and overall. (DOCX)

S3 Appendix Recalled and recognized breast cancer risk factors and symptoms by site. (DOCX)

S4 Appendix Modified Poisson regression showing socio-demographic predictors of higher versus lower breast cancer risk factor and symptom awareness in South Africa. (DOCX)

S5 Appendix Modified Poisson regression showing socio-demographic predictors of higher versus lower breast cancer risk factor and symptom awareness in Uganda. (DOCX)

S6 Appendix Unprompted and prompted breast and cervical cancer lay beliefs. (DOCX)

S7 Appendix Recalled and recognized cervical cancer risk factor and symptom by site. (DOCX)

S8 Appendix Modified Poisson regression showing socio-demographic predictors of higher versus lower cervical cancer risk factor and symptom awareness in South Africa. (DOCX)

S9 Appendix Modified Poisson regression showing socio-demographic predictors of higher versus lower cervical cancer risk factor and symptom awareness in Uganda. (DOCX)

## References

[1] Bray F, Ferlay J, Soerjomataram I, Siegel RL, Torre LA, Jemal A. Global cancer statistics 2018: GLOBOCAN estimates of incidence and mortality worldwide for 36 cancers in 185 countries. CA. Cancer J Clin 2018;68:394–424. 10.3322/caac.21492 30207593

[2] GLOBOCAN. Cancer Today 2018. http://gco.iarc.fr/today/home (accessed 20 Jan 2020).

[3] WHO report on cancer: setting priorities, investing wisely and providing care for all. Geneva: World Health Organization; 2020. Licence: CC BY-NC-SA 3.0 IGO.

[4] Joko-Fru WY, Miranda-Filho A, Soerjomataram I, Egue M, Akele-Akpo M-T, N'da G et al Breast cancer survival in sub-Saharan Africa by age, stage at diagnosis and human development index: a population-based registry study. Int J Cancer 2020;146:1208–18. 10.1002/ijc.32406 31087650 PMC7079125

[5] Sengayi-Muchengeti M, Joko-Fru WY, Miranda-Filho A, Egue M, Akele-Akpo M-T, N'da G et al Cervical cancer survival in sub-Saharan Africa by age, stage at diagnosis and Human Development Index (HDI): A population-based registry study. Int. J. Cancer. 10.1002/ijc.33120 32449157

[6] Ngoma T, Mandeli J, Holland J. Downstaging cancer in rural Africa. Int J Cancer 2015;136:2875–79. 10.1002/ijc.29348 25408458

[7] Macleod U, Mitchell ED, Burgess C, Macdonald S, Ramirez AJ. Risk factors for delayed presentation and referral of symptomatic cancer: evidence for common cancers. Br J Cancer 2009;101: S92–101. 10.1038/sj.bjc.6605398 19956172 PMC2790698

[8] McKenzie F, Zietsman A, Galukande M, Anele A, Adisa C, Parham G et al Drivers of advanced stage at breast cancer diagnosis in the multicountry African breast cancer—disparities in outcomes (ABC-DO) study. Int J Cancer 2018;142:1568–79. 10.1002/ijc.31187 29197068 PMC5838525

[9] Akuoko C, Armah E, Sarpong T, Quansah DY, Amankwaa I, Boateng D. Barriers to early presentation and diagnosis of breast cancer among African women living in sub-Saharan Africa. PLoS ONE 2017;12:e0171024 10.1371/journal.pone.0171024 28192444 PMC5305236

[10] McCutchan GM, Wood F, Edwards A, Richards R, Brain KE. Influences of cancer symptom knowledge, beliefs and barriers on cancer symptom presentation in relation to socioeconomic deprivation: a systematic review. BMC Cancer 2015;15:1000 10.1186/s12885-015-1972-8 26698112 PMC4688960

[11] Shahab L, McGowan JA, Waller J, Smith SG. Prevalence of beliefs about actual and mythical causes of cancer and their association with socio-demographic and health-related characteristics: findings from a cross-sectional survey in England. Eur J Cancer 2018;103:308–16. 10.1016/j.ejca.2018.03.029 29705530 PMC6202672

[12] Devi BCR, Tang TS, Corbex M. Reducing by half the percentage of late-stage presentation for breast and cervix cancer over 4 years: a pilot study of clinical downstaging in Sarawak, Malaysia. Ann Oncol 2007;18:1172–6. 10.1093/annonc/mdm105 17434897

[13] Muthoni A, Miller AN. An Exploration of rural and urban Kenyan women’s knowledge and attitudes regarding breast cancer and breast cancer early detection measures. Health Care Women Int 2010;31:801–16. 10.1080/07399331003628453 20677038

[14] Moodley J, Cairncross L, Naiker T, Constant D. From symptom discovery to treatment—women’s pathways to breast cancer care: a cross-sectional study. BMC Cancer 2018;18:312 10.1186/s12885-018-4219-7 29562894 PMC5863383

[15] Moodley J, Scott SE, Mwaka AD, Constant D, Githaiga JN, Stewart TS et al Development and validation of the African Women Awareness of CANcer (AWACAN) tool for breast and cervical cancer. PLoS ONE 2019;14:e0220545 10.1371/journal.pone.0220545 31386684 PMC6684059

[16] Uganda Bureau of Statistics. 2019 Statistical Abstract. Kampala Uganda https://www.ubos.org/wp-content/uploads/publications/01_20202019_Statistical_Abstract_-Final.pdf (accessed 18 August 2020)

[17] Statistics South Africa. Mid-year population estimates 2019. Statistical release P0302. 2019. Pretoria, Republic of South Africa. http://www.statssa.gov.za/publications/P0302/P03022019.pdf (accessed 18 August 2020)

[18] National Department of Health (NDoH), Statistics South Africa (Stats SA), South African Medical Research Council (SAMRC), and ICF. 2019 South Africa Demographic and Health Survey 2016. Pretoria, South Africa, and Rockville, Maryland, USA: NDoH, Stats SA, SAMRC, and ICF.

[19] African Women Awareness of Cancer (AWACAN). www.awacan.online (accessed 3 February 2020).

[20] Ataguba J, Akazili J, McIntyre D. Socioeconomic-related health inequality in South Africa: evidence from general household surveys. Int J Equity Health 2011;10:48 10.1186/1475-9276-10-48 22074349 PMC3229518

[21] Ali-Risasi C, Mulumba P, Verdonck K, Vanden Broeck D, Praet M. Knowledge, attitude and practice about cancer of the uterine cervix among women living in Kinshasa, the Democratic Republic of Congo. BMC Womens Health. 2014;14(1):30 10.1186/1472-6874-14-30 24548698 PMC3937079

[22] Althobaiti A, Jradi H. Knowledge, attitude, and perceived barriers regarding colorectal cancer screening practices and risk factors among medical students in Saudi Arabia. BMC Med Educ. 2019;19(1):421 10.1186/s12909-019-1857-7 31727029 PMC6854663

[23] Dey S, Mishra A, Govil J, Dhillon PK. Breast Cancer Awareness at the Community Level among Women in Delhi, India. Asian Pac J Cancer Prev. 2015;16(13):5243–5251. 10.7314/apjcp.2015.16.13.5243 26225660

[24] Waller J, McCaffery K, Wardle J. Measuring cancer knowledge: comparing prompted and unprompted recall. Br J Psychol 2004;95:219–34. 10.1348/000712604773952430 15142303

[25] Low EL, Simon AE, Lyons J, Romney-Alexander D, Waller J. What do British women know about cervical cancer symptoms and risk factors? Eur J Cancer 2012;48:3001–8. 10.1016/j.ejca.2012.05.004 22683170

[26] Naanyu V. Lay perceptions of breast cancer in Western Kenya. World J Clin Oncol 2015;6:147–55. 10.5306/wjco.v6.i5.147 26468451 PMC4600189

[27] Koo MM, von Wagner C, Abel GA, McPhail S, Rubin GP, Lyratzopolous. Typical and atypical presenting symptoms of breast cancer and their associations with diagnostic intervals: evidence from a national audit of cancer diagnosis. Cancer Epidemiol 2017;48:140–6. 10.1016/j.canep.2017.04.010 28549339 PMC5482318

[28] Jones C, Maben J, Jack RH, Davies EA, Forbes LJ, Lucas G et al A systematic review of barriers to early presentation and diagnosis with breast cancer among black women. BMJ Open 2014;1:2. 10.1136/bmjopen-2013-004076 PMC3927711 24523424

[29] South African National Department of Health. Cervical Cancer Prevention and Control Policy. Pretoria: NDOH, 2017

[30] https://www.afro.who.int/news/uganda-launches-human-papillomavirus-vaccine (accessed 10 June 2020)

[31] Geremew AB, Gelagay AA, Azale T. Comprehensive knowledge on cervical cancer, attitude towards its screening and associated factors among women aged 30–49 years in Finote Selam town, northwest Ethiopia. Reprod Health 2018;15:29 10.1186/s12978-018-0471-1 29444689 PMC5813403

[32] Gatumo M, Gacheri S, Sayed A-R, Scheibe A. Women’s knowledge and attitudes related to cervical cancer and cervical cancer screening in Isiolo and Tharaka Nithi counties, Kenya: a cross-sectional study. BMC Cancer 2018;18:745 10.1186/s12885-018-4642-9 30021564 PMC6052645

[33] Stein L, Urban MI, O’Connell D, Yu XQ, Beral V, Newton R et al The spectrum of human immunodeficiency virus-associated cancers in a South African black population: results from a case-control study, 1995–2004. Int J Cancer 2008;122:2260–5. 10.1002/ijc.23391 18241034

[34] Moodley JR, Hoffman M, Carrara H, Allan BR, Cooper DD, Rosenberg R et al HIV and pre-neoplastic and neoplastic lesions of the cervix in South Africa: a case-control study. BMC Cancer 2006;6:135 10.1186/1471-2407-6-135 16719902 PMC1481580

[35] Robbins HA, Strickler HD, Massad LS, Pierce CB, Darragh TM, Minkoff H et al Cervical cancer screening intervals and management for women living with HIV: a risk benchmarking approach. AIDS. 2017;31(7):1035–1044. 10.1097/QAD.0000000000001450 28323758 PMC5531443

[36] Pillay AL. Rural and urban South African women's awareness of cancers of the breast and cervix. Ethn Health. 2002;7(2):103–114. 10.1080/1355785022000038588 12511197

[37] Worldbank. South Africa: overview 2020. www.worldbank.org/en/country/southafrica/overview (accessed 3 February 2020).

[38] Tenkorang EY, Gyimah SO, Maticka-Tyndale E, Adeji J. Superstition, witchcraft and HIV prevention in sub-Saharan Africa: the case of Ghana. Cult Health Sex 2011;13:1001–14. 10.1080/13691058.2011.592218 21714753

[39] Mwaka AD, Orach CG, Were EM, Lyratzopoulos G, Wabinga H, Roland M. Awareness of cervical cancer risk factors and symptoms: cross-sectional community survey in post-conflict northern Uganda. Health Expect. 2016;19(4):854–867. 10.1111/hex.12382 26205470 PMC4957614

[40] McFarland DM. Beliefs about the causes of cervical cancer in Botswana: implications for nursing. International Nursing Review. 2009; 56: 426–432. 10.1111/j.1466-7657.2009.00742.x 19930070

[41] Makurirofa L, Mangwiro P, James V, Milanzi A, Mavu J, Nyamuranga M et al Women's knowledge, attitudes and practices (KAP) relating to breast and cervical cancers in rural Zimbabwe: a cross sectional study in Mudzi District, Mashonaland East Province. BMC Public Health. 2019;19(1):109 10.1186/s12889-018-6333-5 30678659 PMC6345058

[42] Hvidberg L, Pedersen AF, Wulff CN, Vedsted P. Cancer awareness and socio-economic position: results from a population-based study in Denmark. BMC Cancer 2014;14:581 10.1186/1471-2407-14-581 25108301 PMC4138385

