# Supplementary material for: Mapping awareness of breast and cervical cancer risk factors, symptoms and lay beliefs in Uganda and South Africa
Source: PLoS One. 2020 Oct 22;15(10):e0240788. doi: 10.1371/journal.pone.0240788 (PMC7580973; doi:10.1371/journal.pone.0240788)
Supplement: S2 Appendix — (DOCX) [file pone.0240788.s002.docx]

**S2 Appendix: Participants assets by site, country and overall (n=1758)**

|  | **South Africa** | | **Uganda** | | **Total** |
| --- | --- | --- | --- | --- | --- |
|  | **Urban** | **Rural** | **Urban** | **Rural** |  |
|  | **n (%)** | **n (%)** | **n (%)** | **n (%)** | **n (%)** |
| **Do you have electricity or a generator or solar panel*** | **n=444** | **n=428** | **n=458** | **n=426** | **n=1756** |
| Yes | 422 (95.1) | 396 (92.5) | 264 (57.6) | 172 (40.4) | 1254 (71.4) |
| No | 22 (4.9) | 32 (7.5) | 194 (42.4) | 254 (59.6) | 502 (28.6) |
| **Do you have tap water in your house, compound or property*** | **n=445** | **n=428** | **n=458** | **n=427** | **n=1758** |
| Yes | 363 (81.6) | 18 (4.2) | 128 (27.9) | 11(2.6) | 520 (29.6) |
| No | 82 (18.4) | 410 (95.8) | 330 (72.1) | 416 (97.4) | 1238 (70.4) |
| **Do you have any type of toilet in your house, compound or property** | **n=445** | **n=428** | **n=458** | **n=427** | **n=1758** |
| Yes | 359 (80.7) | 392 (91.6) | 445 (97.2) | 382 (89.5) | 1578 (89.8) |
| No | 86 (19.3) | 36 (8.4) | 13 (2.8) | 45 (10.5) | 180 (10.2) |
| **Do you or does anyone living with you have a radio*** | **n=444** | **n=427** | **n=457** | **n=427** | **n=1755** |
| Yes | 428 (96.4) | 231 (54.1) | 281 (61.5) | 214 (50.1) | 1154 (65.8) |
| No | 16 (3.6) | 196 (45.9) | 176 (38.5) | 213 (49.9) | 601 (34.2) |
| **Do you or does anyone living with you have a television*** | **n=445** | **n=428** | **n=458** | **n=426** | **n=1757** |
| Yes | 405 (91.0) | 303 (70.8) | 161 (35.2) | 20 (4.7) | 889 (50.6) |
| No | 40 (9.0) | 125 (29.2) | 297 (64.9) | 406 (95.3) | 868 (49.4) |
| **Do you or does anyone living with you have internet access on a computer, a laptop or a mobile phone*** | **n=444** | **n=426** | **n=451** | **n=427** | **n=1748** |
| Yes | 405 (91.2) | 159 (37.3) | 215 (47.7) | 58 (13.6) | 837 (47.9) |
| No | 39 (8.8) | 267 (62.7) | 236 (52.3) | 369 (86.4) | 911 (52.1) |
| **In what type of dwelling or housing do you live** | **n=445** | **n=428** | **n=458** | **n=427** | **n=1758** |
| Brick house/apartment | 273 (61.4) | 356 (83.2) | 258 (56.3) | 32 (7.5) | 919 (52.3) |
| Informal/traditional dwelling | 172 (38.6) | 72 (16.8) | 200 (43.7) | 395 (92.5) | 839 (47.7) |

*variables included in the Asset Index
