# Supplementary material for: Mapping awareness of breast and cervical cancer risk factors, symptoms and lay beliefs in Uganda and South Africa
Source: PLoS One. 2020 Oct 22;15(10):e0240788. doi: 10.1371/journal.pone.0240788 (PMC7580973; doi:10.1371/journal.pone.0240788)
Supplement: S3 Appendix — (DOCX) [file pone.0240788.s003.docx]

**S3 Appendix: Recalled and recognized breast cancer risk factors and symptoms by site**

|  | **South Africa Urban** | | **South Africa Rural** | | **Uganda Urban** | | **Uganda Rural** | | **Total** | |
| --- | --- | --- | --- | --- | --- | --- | --- | --- | --- | --- |
|  | **Recalled** | **Recognized** | **Recalled** | **Recognized** | **Recalled** | **Recognized** | **Recalled** | **Recognized** | **Recalled** | **Recognized** |
|  | **n (%)** | **n (%)** | **n (%)** | **n (%)** | **n (%)** | **n (%)** | **n (%)** | **n (%)** | **n (%)** | **n (%)** |
| **Risk factor (13)** | **n=406** | **n=406** | **n=342** | **n=342** | **n=445** | **n=445** | **n=403** | **n=403** | **n=1596** | **n=1596** |
| Having family member with breast cancer | 17 (4.2) | 374 (92.1) | 3 (0.9) | 123 (36.0) | 27 (6.1) | 147 (33.0) | 3 (0.7) | 146 (36.2) | 50 (3.1) | 790 (49.5) |
| Having had breast cancer previously | 1 (0.3) | 353 (86.9) | 0 (0.0) | 119 (34.8) | 0 (0.0) | 240 (53.9) | 0 (0.0) | 133 (33.0) | 1 (0.1) | 845 (52.9) |
| Using hormonal Family Planning methods | 1 (0.3) | 212 (52.2) | 0 (0.0) | 158 (46.2) | 7 (1.6) | 232 (52.1) | 2 (0.5) | 178 (44.2) | 10 (0.6) | 780 (48.9) |
| Drinking > 1 helping of alcohol a day | 39 (9.6) | 194 (47.8) | 3 (0.9) | 138 (40.4) | 3 (0.7) | 144 (32.4) | 1 (0.3) | 181 (44.9) | 46 (2.9) | 657 (41.2) |
| Using hormone replacement therapy | 0 (0.0) | 191 (47.0) | 0 (0.0) | 78 (22.8) | 0 (0.0) | 229 (51.5) | 0 (0.0) | 131 (32.5) | 0 (0.0) | 629 (39.4) |
| Having menopause >55 years of age | 0 (0.0) | 162 (39.9) | 0 (0.0) | 112 (32.7) | 0 (0.0) | 152 (34.2) | 0 (0.0) | 167 (41.4) | 0 (0.0) | 593 (37.2) |
| Being overweight | 2 (0.5) | 151 (37.2) | 0 (0.0) | 135 (39.5) | 4 (0.9) | 186 (41.8) | 0 (0.0) | 241 (59.8) | 6 (0.4) | 713 (44.7) |
| Having no children at all | 3 (0.7) | 141 (34.7) | 0 (0.0) | 83 (24.3) | 4 (0.9) | 141 (31.7) | 0 (0.0) | 161 (40.0) | 7 (0.4) | 526 (37.2) |
| Growing old | 3 (0.7) | 139 (34.2) | 0 (0.0) | 159 (46.5) | 2 (0.5) | 115 (25.8) | 0 (0.0) | 91 (22.6) | 5 (0.3) | 504 (31.6) |
| Starting periods <11 years of age | 0 (0.0) | 130 (32.0) | 0 (0.0) | 111 (32.4) | 0 (0.0) | 105 (23.6) | 0 (0.0) | 155 (38.5) | 0 (0.0) | 501 (31.4) |
| Having first child after 30 years of age | 0 (0.0) | 129 (31.8) | 1 (0.3) | 79 (23.1) | 1 (0.2) | 127 (28.5) | 0 (0.0) | 138 (34.2) | 2 (0.1) | 473 (29.6) |
| Not breastfeeding | 8 (2.0) | 101 (24.9) | 2 (0.6) | 92 (26.9) | 21(4.7) | 216 (48.5) | 4 (1.0) | 178 (44.2) | 35 (2.2) | 587 (36.8) |
| Doing little physical activity | 1 (0.3) | 89 (21.9) | 0 (0.0) | 50 (14.6) | 0 (0.0) | 130 (29.2) | 0 (0.0) | 115 (28.5) | 1 (0.1) | 384 (24.1) |
| **At least 1 risk factor** | 66 (16.3) | 405 (99.8) | 9 (2.6) | 319 (93.3) | 54 (12.1) | 420 (94.4) | 10 (2.5) | 375 (93.1) | 139 (8.7) | 1519 (95.2) |
| **Median recognized risk factor score**  **(Interquartile Range)** |  | 5  (3-9) |  | 4  (2-6) |  | 5  (2-7) |  | 5  (3-7) |  | 4  (3-7) |
| **Symptom (15)** | **n=406** | **n=445** | **n=342** | **n=428** | **n=445** | **n=458** | **n=403** | **n=427** | **n=1596** | **n=1758** |
| A lump or thickening in breast | 276 (68.0) | 435 (97.8) | 192 (56.1) | 391 (91.4) | 157(35.3) | 420 (91.7) | 91 (22.6) | 350 (82.0) | 716 (44.9) | 1596 (90.8) |
| Pulling in of nipple | 2 (0.5) | 432 (97.1) | 1(0.3) | 335 (78.3) | 7 (1.6) | 387 (84.5) | 3 (0.7) | 344 (80.6) | 13 (0.8) | 1498 (85.2) |
| Bleeding from nipple | 18 (4.4) | 430 (96.6) | 1 (0.3) | 373 (87.1) | 17 (3.8) | 423 (92.4) | 18 (4.5) | 380 (89.0) | 54 (3.4) | 1606 (91.4) |
| Change in position of nipple | 1 (0.3) | 429 (96.4) | 0(0.0) | 324 (75.7) | 0 (0.0) | 356 (77.7) | 1 (0.3) | 323 (75.6) | 2 (0.1) | 1432 (81.5) |
| Discharge from nipple | 60 (14.8) | 427 (96.0) | 16(14.7) | 349 (81.5) | 45 (10.1) | 403 (88.0) | 22 (5.5) | 341 (79.9) | 143 (9.0) | 1520 (86.5) |
| Puckering of the breast skin | 1 (0.2) | 426 (95.7) | 1 (0.3) | 328 (76.6) | 4 (0.9) | 418 (91.3) | 8 (2.0) | 363 (85.0) | 14 (0.9) | 1535 (87.3) |
| Change in the size of nipple | 7 (1.7) | 421 (94.6) | 1 (0.3) | 334 (78.0) | 2 (0.5) | 346 (75.5) | 7 (1.7) | 321 (75.2) | 17 (1.1) | 1422 (80.9) |
| Nipple rash | 8 (2.0) | 421 (94.6) | 2 (0.6) | 339 (79.2) | 0 (0.0) | 335 (73.1) | 23 (5.7) | 320 (74.9) | 33 (2.1) | 1415 (80.5) |
| Change in the size of the breast | 96 (23.7) | 419 (94.2) | 33 (9.7) | 334 (78.0) | 184 (41.4) | 360 (78.6) | 142 (35.2) | 333 (78.0) | 455 (28.5) | 1446 (82.3) |
| Change in the shape of nipple | 2 (0.5) | 417 (93.7) | 0 (0.0) | 311 (72.7) | 0 (0.0) | 343 (74.9) | 6 (1.5) | 324 (75.9) | 8 (0.5) | 1395 (79.4) |
| Change in colour of the breast skin | 44 (10.8) | 413 (92.8) | 3 (0.9) | 348 (81.3) | 17 (3.8) | 392 (85.6) | 9 (2.2) | 333 (78.0) | 73 (4.6) | 1486 (84.5) |
| Change in the shape of breast | 10 (2.5) | 411 (92.4) | 0 (0.0) | 326 (76.2) | 4 (0.9) | 350 (76.4) | 3 (0.7) | 314 (73.5) | 17 (1.1) | 1401 (79.7) |
| A lump or thickening under the armpit | 12 (3.0) | 403 (90.6) | 3 (0.9) | 278 (75.0) | 3 (0.7) | 319 (69.7) | 2 (0.5) | 278 (65.1) | 20 (1.3) | 1278 (72.7) |
| Pain in one or both breasts | 93 (22.9) | 401 (90.1) | 57 (16.7) | 345 (80.6) | 225 (50.6) | 419 (91.5) | 157 (39.0) | 380 (89.0) | 532 (33.3) | 1545 (87.9) |
| Pain in the armpit | 2 (0.5) | 369 (82.9) | 1 (0.3) | 250 (58.4) | 1 (0.2) | 285 (62.2) | 2 (0.5) | 298 (69.8) | 6 (0.4) | 1202 (68.4) |
| **At least 1 symptom** | 371 (91.4) | 445 (100) | 237 (69.3) | 421 (98.4) | 353 (79.3) | 456 (99.6) | 284 (70.5) | 284 (70.5) | 1245 (78.0) | 1740 (99.0) |
| **Median recognized symptom score**  **(Interquartile Range)** |  | 15  (14-15) |  | 13  (10-14) |  | 13  (11-15) |  | 13  (10-15) |  | 14  (11-15) |
