# Supplementary material for: Mapping awareness of breast and cervical cancer risk factors, symptoms and lay beliefs in Uganda and South Africa
Source: PLoS One. 2020 Oct 22;15(10):e0240788. doi: 10.1371/journal.pone.0240788 (PMC7580973; doi:10.1371/journal.pone.0240788)
Supplement: S4 Appendix — (DOCX) [file pone.0240788.s004.docx]

**S4 Appendix: Modified Poisson regression showing socio-demographic predictors of higher versus lower breast cancer risk factor and symptom awareness in South Africa**

|  | **Breast cancer risk factors**  n=722 Pseudo R^2^=0.011 Pr >Chi^2^=0.004 | | **Breast cancer symptoms**  n=837 Pseudo R^2^=0.065. Pr >Chi^2^<0.001 | |
| --- | --- | --- | --- | --- |
|  | **Prevalence ratio (95% Confidence interval)** | **p-value** | **Prevalence ratio (95% Confidence interval)** | **p-value** |
| **Location** |  |  |  |  |
| Rural | Referent |  | Referent |  |
| Urban | 1.35 (1.07-1.72) | 0.013 | 2.83 (2.24-3.57) | <0.001 |
|  |  |  |  |  |
| **Age** |  |  |  |  |
| 18-29 | Referent |  | Referent |  |
| 30-49 | 1.28 (1.03-1.58) | 0.023 | 1.11 (0.94-1.32) | 0.210 |
| ≥ 50 | 1.23 (0.94-1.61) | 0.126 | 1.25 (1.00-1.56) | 0.048 |
|  |  |  |  |  |
| **Relationship status** |  |  |  |  |
| Married/Living with a partner | Referent |  | Referent |  |
| No partner/not living with partner | 1.03 (0.87-1.22) | 0.739 | 0.92 (0.79-1.06) | 0.251 |
| Separated/Divorced/Widowed | 0.82 (0.59-1.14) | 0.233 | 0.70 (0.50-1.00) | 0.051 |
|  |  |  |  |  |
| **Highest educational level completed** |  |  |  |  |
| No schooling to primary incomplete | Referent |  | Referent |  |
| Primary complete to secondary incomplete | 0.97 (0.76-1.26) | 0.844 | 1.29 (0.96-1.74) | 0.088 |
| Secondary complete or more | 0.92 (0.70-1.21) | 0.544 | 1.41 (1.04-1.91) | 0.027 |
|  |  |  |  |  |
| **Paid work** |  |  |  |  |
| No | Referent |  | Referent |  |
| Yes | 1.15 (0.95-1.39) | 0.139 | 0.84 (0.71-0.98) | 0.026 |
|  |  |  |  |  |
| **Asset Index** |  |  |  |  |
| Upper tercile | Referent |  | Referent |  |
| Middle tercile | 1.06 (0.86-1.31) | 0.595 | 1.17 (1.00-1.38) | 0.051 |
| Lower tercile | 1.16 (0.91-1.48) | 0.223 | 1.10 (0.86-1.35) | 0.499 |
|  |  |  |  |  |
