# Supplementary material for: Mapping awareness of breast and cervical cancer risk factors, symptoms and lay beliefs in Uganda and South Africa
Source: PLoS One. 2020 Oct 22;15(10):e0240788. doi: 10.1371/journal.pone.0240788 (PMC7580973; doi:10.1371/journal.pone.0240788)
Supplement: S5 Appendix — (DOCX) [file pone.0240788.s005.docx]

**S5 Appendix: Modified Poisson regression showing socio-demographic predictors of higher versus lower breast cancer risk factor and symptom awareness in Uganda**

|  | **Breast cancer risk factors**  n=842 Pseudo R^2^=0.015 Pr > Chi^2^=0.006 | | **Breast cancer symptoms**  n=879 Pseudo R^2^=0.009 Pr > Chi^2^=0.021 | |
| --- | --- | --- | --- | --- |
|  | **Prevalence ratio (95% Confidence interval)** | **p-value** | **Prevalence ratio (95% Confidence interval)** | **p-value** |
| **Location** |  |  |  |  |
| Rural | Referent |  | Referent |  |
| Urban | 1.03 (0.85-1.23) | 0.787 | 1.04 (0.87-1.24) | 0.672 |
|  |  |  |  |  |
| **Age** |  |  |  |  |
| 18-29 | Referent |  | Referent |  |
| 30-49 | 1.10 (0.85-1.23) | 0.309 | 1.37 (1.15-1.63) | 0.001 |
| ≥ 50 | 1.02 (0.80-1.30) | 0.887 | 1.32 (1.04-1.69) | 0.023 |
|  |  |  |  |  |
| **Relationship status** |  |  |  |  |
| Married/Living with a partner | Referent |  | Referent |  |
| No partner/not living with partner | 0.37 (0.19-0.73) | 0.004 | 1.05 (0.75-1.46) | 0.769 |
| Separated/Divorced/Widowed | 1.00 (0.81-1.21) | 0.930 | 0.84 (0.68-1.04) | 0.117 |
|  |  |  |  |  |
| **Highest educational level completed** |  |  |  |  |
| No schooling to primary incomplete | Referent |  | Referent |  |
| Primary complete to secondary incomplete | 0.84 (0.68-1.04) | 0.115 | 1.10 (0.91-1.34) | 0.326 |
| Secondary complete or more | 0.76 (0.56-1.04) | 0.085 | 1.27 (1.00-1.64) | 0.075 |
|  |  |  |  |  |
| **Paid work** |  |  |  |  |
| No | Referent |  | Referent |  |
| Yes | 0.95 (0.79-1.14) | 0.577 | 0.87 (0.73-1.04) | 0.118 |
|  |  |  |  |  |
| **Asset Index** |  |  |  |  |
| Upper tercile | Referent |  | Referent |  |
| Middle tercile | 1.12 (0.91-1.40) | 0.288 | 1.14 (0.92-1.40) | 0.227 |
| Lower tercile | 1.10 (0.86-1.41) | 0.430 | 0.96 (0.75-1.23) | 0.749 |
