# Supplementary material for: Mapping awareness of breast and cervical cancer risk factors, symptoms and lay beliefs in Uganda and South Africa
Source: PLoS One. 2020 Oct 22;15(10):e0240788. doi: 10.1371/journal.pone.0240788 (PMC7580973; doi:10.1371/journal.pone.0240788)
Supplement: S6 Appendix — (DOCX) [file pone.0240788.s006.docx]

**S6 Appendix: Unprompted and prompted breast and cervical cancer lay beliefs**

|  | **South Africa** | | | | **Uganda** | | | | **Total** | |
| --- | --- | --- | --- | --- | --- | --- | --- | --- | --- | --- |
|  | **Urban** | | **Rural** | | **Urban** | | **Rural** | |  | |
|  | **Unprompted** | **Prompted** | **Unprompted** | **Prompted** | **Unprompted** | **Prompted** | **Unprompted** | **Prompted** | **Unprompted** | **Prompted** |
|  | **n (%)** | **n (%)** | **n (%)** | **n (%)** | **n (%)** | **n (%)** | **n (%)** | **n (%)** | **n (%)** | **n (%)** |
| **Breast Cancer** | | | | | | | | | | |
| **Risk Lay beliefs (6)** | **n=406** | **n=406** | **n=342** | **n=342** | **n=445** | **n=445** | **n=403** | **n=403** | **n=1596** | **n=1596** |
| Putting money in one’s bra | 209 (51.5) | 397 (97.8) | 38 (11.1) | 291 (85.1) | 46 (10.3) | 378 (84.9) | 44 (10.9) | 335 (83.1) | 337 (21.1) | 1401 (87.8) |
| Wearing a bra all the time | 25 (6.2) | 377 (92.9) | 2 (0.6) | 238 (69.6) | 31 (7.0) | 388 (87.2) | 14 (3.5) | 340 (84.4) | 72 (4.5) | 1343 (84.2) |
| Wearing a tight bra | 45 (11.1) | 371 (91.4) | 1 (0.3) | 190 (55.6) | 60 (13.5) | 387 (87.0) | 14 (3.5) | 331 (82.1) | 120 (7.5) | 1279 (80.1) |
| Putting mobile phone in bra | 53 (13.1) | 371 (91.4) | 12 (3.5) | 246 (71.9) | 12 (2.7) | 364 (81.8) | 3 (0.7) | 281 (69.3) | 80 (5.0) | 1262 (79.1) |
| Being exposed to dirty air/water | 5 (1.2) | 232 (57.1) | 1 (0.3) | 177 (51.6) | 1 (0.2) | 273 (61.4) | 0 (0.0) | 303 (75.2) | 7 (0.4) | 985 (61.7) |
| Being bewitched | 11 (2.7) | 128 (31.5) | 1 (0.3) | 88 (25.7) | 0 (0.0) | 65 (14.6) | 0 (0.0) | 74 (18.4) | 12 (0.8) | 355 (22.2) |
| **Symptom Lay Beliefs** | **n=406** | **n=445** | **n=342** | **n=428** | **n=452** | **n=458** | **n=411** | **n=427** | **n=748** | **n=1758** |
| Fever | 0 (0.0) | 90 (20.2) | 0 (0.0) | 42 (9.8) | 9 (2.0) | 223 (48.7) | 1 (0.3) | 228 (53.4) | 10 (0.6) | 583 (33.2) |
|  |  |  |  |  |  |  |  |  |  |  |
| **Cervical Cancer** | | | | | | | | | | |
| **Risk Lay Beliefs** | **n=365** | **n=366** | **n=342** | **n=342** | **n=450** | **n=452** | **n=411** | **n=411** | **n=1568** | **n=1571** |
| Inserting herbs/creams/objects into the vagina | 40 (11.0) | 355 (97.0) | 10 (2.9) | 281 (82.2) | 21 (4.7) | 375 (83.0) | 5 (1.2) | 331 (80.5) | 76 (4.9) | 1342 (85.4) |
| Poor personal hygiene | 155 (42.5) | 354 (96.7) | 18 (5.3) | 294 (86.0) | 184 (40.9) | 420 (92.9) | 172 (41.9) | 391 (95.1) | 529 (33.7) | 1459 (92.9) |
| Being bewitched | 3 (0.8) | 121 (33.1) | 1 (0.3) | 108 (31.6) | 2 (0.4) | 58 (12.8) | 0 (0.0) | 63 (15.3) | 6 (0.4) | 350 (22.3) |
| Using condoms | 10 (2.8) | 106 (29.0) | 3 (0.9) | 59 (17.3) | 35 (7.8) | 244 (54.0) | 4 (1.0) | 195 (47.5) | 52 (3.3) | 604 (38.5) |
| **Symptom Lay Beliefs** | **n=365** | **n=445** | **n=342** | **n=428** | **n=450** | **n=458** | **n=412** | **n=427** | **n=1569** | **n=1758** |
| Itching in vagina | 39 (10.7) | 399 (89.7) | 10 (2.9) | 315 (73.6) | 41 (9.1) | 299 (65.3) | 36 (8.7) | 271 (63.5) | 126 (8.0) | 1284 (73.0) |
