# Supplementary material for: Mapping awareness of breast and cervical cancer risk factors, symptoms and lay beliefs in Uganda and South Africa
Source: PLoS One. 2020 Oct 22;15(10):e0240788. doi: 10.1371/journal.pone.0240788 (PMC7580973; doi:10.1371/journal.pone.0240788)
Supplement: S7 Appendix — (DOCX) [file pone.0240788.s007.docx]

**S7 Appendix: Recalled and recognized cervical cancer risk factors and symptoms by site**

|  | **South Africa Urban** | | **South Africa Rural** | | **Uganda Urban** | | **Uganda Rural** | | **Total** | |
| --- | --- | --- | --- | --- | --- | --- | --- | --- | --- | --- |
|  | **Recalled** | **Recognized** | **Recalled** | **Recognized** | **Recalled** | **Recognized** | **Recalled** | **Recognized** | **Recalled** | **Recognized** |
|  | **n (%)** | **n (%)** | **n (%)** | **n (%)** | **n (%)** | **n (%)** | **n (%)** | **n (%)** | **n (%)** | **n (%)** |
| **Risk Factors (11)** | **n=365** | **n=366** | **n=342** | **n=342** | **n=450** | **n=452** | **n=411** | **n=411** | **n=1568** | **n=1571** |
| Having many sexual partners | 65 (17.8) | 342 (93.4) | 11 (3.2) | 284 (83.0) | 149 (33.1) | 410 (90.7) | 74 (18.0) | 389 (94.7) | 299 (19.1) | 1425 (90.7) |
| Having unprotected sex | 105 (28.8) | 340 (92.9) | 19 (5.6) | 271 (79.2) | 3 (0.7) | 310 (68.6) | 4 (1.0) | 269 (65.5) | 131 (8.4) | 1190 (75.8) |
| Not going for regular screening | 7 (1.9) | 337 (92.1) | 1 (0.3) | 251 (73.4) | 5 (1.1) | 281 (62.2) | 1 (0.2) | 285 (69.3) | 14 (0.9) | 1154 (73.5) |
| Other sexually transmitted diseases | 55 (15.1) | 306 (83.6) | 40 (11.7) | 275 (80.4) | 27 (6.0) | 352 (77.8) | 17 (4.1) | 305 (74.2) | 139 (8.9) | 1238 (78.8) |
| Sexual partner is not circumcised | 6 (1.6) | 301 (82.2) | 0 (0.0) | 187 (54.7) | 6 (1.3) | 251 (55.5) | 3 (0.7) | 263 (64.0) | 15 (1.0) | 1002 (63.8) |
| Having sex at a young age | 5 (1.4) | 288 (78.7) | 2 (0.6) | 248 (72.5) | 25 (5.6) | 360 (79.7) | 23 (5.6) | 357 (86.9) | 55 (3.5) | 1253 (79.8) |
| Getting an HPV infection | 2 (0.6) | 285 (77.9) | 0 (0.0) | 238 (69.6) | 4 (0.9) | 354 (78.3) | 0 (0.0) | 321 (78.1) | 6 (0.4) | 1198 (76.3) |
| Using family planning for >5 years | 1 (0.3) | 256 (70.0) | 2 (0.6) | 190 (55.6) | 11 (2.4) | 323(71.5) | 7 (1.7) | 313 (76.2) | 21 (1.3) | 1082 (68.9) |
| HIV/AIDS | 2 (0.6) | 253 (69.1) | 0 (0.0) | 161 (47.1) | 3 (0.7) | 222 (49.1) | 0 (0.0) | 200 (48.7) | 5 (0.3) | 836 (53.2) |
| Smoking at all | 7 (1.2) | 212 (57.9) | 14 (4.1) | 284 (83.0) | 3 (0.7) | 193 (42.7) | 1 (0.2) | 229 (55.7) | 25 (1.6) | 918 (53.4) |
| Giving birth to ≥ 3 children | 1 (0.3) | 155 (42.4) | 4 (1.2) | 86 (25.2) | 13 (2.9) | 126 (27.9) | 5 (1.2) | 162 (39.4) | 23 (1.5) | 529 (33.7) |
| **At least 1 risk factor** | 203 (55.6) | 365 (99.7) | 86 (25.2) | 335 (98.0) | 193 (42.9) | 450 (99.6 ) | 116 (28.2) | 407 (99.0) | 598 (38.1) | 1557 (99.1) |
| **Median recognized risk factor score (Interquartile Range)** |  | 9  (7-10) |  | 8  (6-9) |  | 7  (5-9) |  | 8  (6-9) |  | 8  (6-9) |
|  |  |  |  |  |  |  |  |  |  |  |
| **Symptoms (11)** | **n=365** | **n=445** | **n=342** | **n=428** | **n=450** | **n=458** | **n=412** | **n=427** | **n=1569** | **n=1758** |
| Smelly vaginal discharge | 167 (45.8) | 419 (94.2) | 33 (9.7) | 366 (85.5) | 89 (19.8) | 424 (92.6) | 43 (10.4) | 372 (87.1) | 332 (21.2) | 1581 (89.9) |
| Bleeding between periods | 113 (31.0) | 408 (92.0) | 77 (22.5) | 357 (83.4) | 143 (31.8) | 391 (85.4) | 100 (24.3) | 362 (84.8) | 433 (27.6) | 1518 (86.4) |
| Blood in urine or stool | 2 (0.6) | 409 (91.9) | 1 (0.3) | 314 (73.4) | 0 (0.0) | 305 (66.6) | 1(0.2) | 278 (65.1) | 4 (0.3) | 1306 (74.3) |
| Periods heavier/longer than usual | 18 (4.9) | 405 (91.0) | 17 (5.0) | 360 (84.1) | 18 (4.0) | 339 (74.0) | 15 (3.6) | 312 (73.1) | 68 (4.3) | 1416 (80.6) |
| Vaginal bleeding after menopause | 3 (0.8) | 399 (89.7) | 5 (1.5) | 370 (86.5) | 9 (2.0) | 406 (88.7) | 3 (0.7) | 361 (84.5) | 20 (1.3) | 1536 (87.4) |
| Bleeding during or after sex | 4 (1.1) | 398 (89.4) | 0 (0.0) | 305 (71.3) | 8 (1.8) | 384(83.8) | 0 (0.0) | 342 (80.1) | 12 (0.8) | 1429 (81.3) |
| Lower abdominal/pelvic pain | 54 (14.8) | 394 (88.5) | 38 (11.1) | 349 (81.5) | 167 (37.1) | 417 (91.1) | 119 (28.9) | 371 (86.9) | 378 (24.1) | 1531 (87.1) |
| Pain during sex | 13 (3.6) | 371 (83.4) | 1 (0.3) | 298 (69.6) | 22 (4.9) | 380 (83.0) | 9 (2.2) | 341 (79.9) | 45 (2.9) | 1390 (79.1) |
| Lower back pain | 7 (1.9) | 357 (80.2) | 0 (0.0) | 263 (61.5) | 3 (0.7) | 355 (77.5) | 11 (2.7) | 314 (73.5) | 21 (1.3) | 1289 (73.3) |
| Unexplained weight loss | 11 (3.0) | 264 (59.3) | 5 (1.5) | 236 (55.1) | 7 (1.6) | 235 (51.3) | 3 (0.7) | 214 (50.1) | 26 (1.7) | 949 (54.0) |
| Persistent diarrhoea | 2 (0.6) | 162 (36.4) | 0 (0.0) | 118 (27.6) | 0 (0.0) | 157 (34.3) | 0 (0.0) | 178 (41.7) | 2 (0.1) | 615 (35.0) |
| **At least 1 risk symptom** | 267 (73.2) | 438 (98.4) | 153 (44.7) | 420 (98.1) | 288 (64.0) | 452 (98.7) | 197 (47.8) | 411 (96.3) | 905 (57.7) | 1721 (97.9) |
| **Median recognized symptom score (Interquartile Range)** |  | 9  (8-11) |  | 6  (8-10) |  | 9  (7-10) |  | 9  (6-10) |  | 9  (7-10) |
