# Supplementary material for: Mapping awareness of breast and cervical cancer risk factors, symptoms and lay beliefs in Uganda and South Africa
Source: PLoS One. 2020 Oct 22;15(10):e0240788. doi: 10.1371/journal.pone.0240788 (PMC7580973; doi:10.1371/journal.pone.0240788)
Supplement: S8 Appendix — (DOCX) [file pone.0240788.s008.docx]

**S8 Appendix: Modified Poisson regression showing socio-demographic predictors of higher versus lower cervical cancer risk factor and symptom awareness in South Africa**

|  | **Cervical cancer risk factors**  n=685 Pseudo R^2^=0.013 Pr > Chi^2^=0.006 | | **Cervical cancer symptoms**  n=837 Pseudo R^2^=0.023 Pr > Chi^2^ <0.001 | |
| --- | --- | --- | --- | --- |
|  | **Prevalence ratio (95% Confidence interval)** | **p-value** | **Prevalence ratio (95% Confidence interval)** | **p-value** |
| **Location** |  |  |  |  |
| Rural | Referent |  | Referent |  |
| Urban | 1.55 (1.22-1.99) | <0.001 | 1.79 (1.41-2.27) | <0.001 |
|  |  |  |  |  |
| **Age** |  |  |  |  |
| 18-29 | Referent |  | Referent |  |
| 30-49 | 1.16 (0.94-1.43) | 0.172 | 1.37 (1.10-1.71) | 0.005 |
| ≥ 50 | 1.20 (0.92-1.60) | 0.173 | 1.47 (1.13-1.92) | 0.004 |
|  |  |  |  |  |
| **Relationship status** |  |  |  |  |
| Married/Living with a partner | Referent |  | Referent |  |
| No partner/not living with partner | 0.98 (0.82-1.17) | 0.806 | 0.95 (0.80-1.13) | 0.580 |
| Separated/Divorced/Widowed | 0.70 (0.50-1.00) | 0.051 | 0.81 (0.59-1.10) | 0.172 |
|  |  |  |  |  |
| **Highest educational level completed** |  |  |  |  |
| No schooling to primary incomplete | Referent |  | Referent |  |
| Primary complete to secondary incomplete | 0.96 (0.74-1.24) | 0.739 | 0.98 (0.77-1.25) | 0.897 |
| Secondary complete or more | 0.96 (0.73-1.30) | 0.772 | 0.79 (0.60-1.04) | 0.099 |
|  |  |  |  |  |
| **Paid work** |  |  |  |  |
| No | Referent |  | Referent |  |
| Yes | 1.02 (0.84-1.23) | 0.879 | 1.14 (0.94-1.38) | 0.180 |
|  |  |  |  |  |
| **Asset Index** |  |  |  |  |
| Upper tercile | Referent |  | Referent |  |
| Middle tercile | 1.04 (0.84-1.29) | 0.727 | 1.18 (0.96-1.45) | 0.118 |
| Lower tercile | 1.23 (0.96-1.57) | 0.107 | 1.34 (1.05-1.70) | 0.018 |
