# Supplementary material for: Mapping awareness of breast and cervical cancer risk factors, symptoms and lay beliefs in Uganda and South Africa
Source: PLoS One. 2020 Oct 22;15(10):e0240788. doi: 10.1371/journal.pone.0240788 (PMC7580973; doi:10.1371/journal.pone.0240788)
Supplement: S9 Appendix — (DOCX) [file pone.0240788.s009.docx]

**S9 Appendix: Modified Poisson regression showing socio-demographic predictors of higher versus lower cervical cancer risk factor and symptom awareness in Uganda**

|  | **Cervical cancer risk factors**  n=858 Pseudo R^2^=0.015 Pr > Chi^2^=0.002 | | **Cervical cancer symptoms**  n=879 Pseudo R^2^=0.007 Pr > Chi^2^=0.197 | |
| --- | --- | --- | --- | --- |
|  | **Prevalence ratio (95% Confidence interval)** | **p-value** | **Prevalence ratio (95% Confidence interval)** | **p-value** |
| **Location** |  |  |  |  |
| Rural | Referent |  | Referent |  |
| Urban | 0.98 (0.81-1.19) | 0.838 | 1.08 (0.89-1.31) | 0.421 |
|  |  |  |  |  |
| **Age** |  |  |  |  |
| 18-29 | Referent |  | Referent |  |
| 30-49 | 1.06 (0.88-1.28) | 0.539 | 1.05 (0.88-1.27) | 0.575 |
| ≥ 50 | 1.02 (0.79-1.32) | 0.882 | 0.94 (0.72-1.23) | 0.656 |
|  |  |  |  |  |
| **Relationship status** |  |  |  |  |
| Married/Living with a partner | Referent |  | Referent |  |
| No partner/not living with partner | 0.87 (0.57-1.31) | 0.502 | 0.60 (0.37-0.98) | 0.043 |
| Separated/Divorced/Widowed | 0.89 (0.71-1.12) | 0.328 | 0.87 (0.69-1.09) | 0.224 |
|  |  |  |  |  |
| **Highest educational level completed** |  |  |  |  |
| No schooling to primary incomplete | Referent |  | Referent |  |
| Primary complete to secondary incomplete | 0.89 (0.72-1.10) | 0.267 | 1.02 (0.83-1.25) | 0.881 |
| Secondary complete or more | 0.66 (0.46-0.96) | 0.029 | 0.85 (0.62-1.17) | 0.321 |
|  |  |  |  |  |
| **Paid work** |  |  |  |  |
| No | Referent |  | Referent |  |
| Yes | 0.82 (0.67-0.99) | 0.042 | 0.86 (0.71-1.03) | 0.101 |
|  |  |  |  |  |
| **Asset Index** |  |  |  |  |
| Upper tercile | Referent |  | Referent |  |
| Middle tercile | 1.27 (1.00-1.60) | 0.045 | 1.10 (0.89-1.37) | 0.385 |
| Lower tercile | 1.12 (0.86-1.47) | 0.409 | 0.95 (0.73-1.23) | 0.696 |
