# Supplementary material for: Mapping awareness of breast and cervical cancer risk factors, symptoms and lay beliefs in Uganda and South Africa
Source: PLoS One. 2020 Oct 22;15(10):e0240788. doi: 10.1371/journal.pone.0240788 (PMC7580973; doi:10.1371/journal.pone.0240788)
Supplement: Attachment — Submitted filename: Reviewer comments.docx [file pone.0240788.s010.docx]

**General comments:**

The manuscript titled Mapping breast and cervical cancer awareness in Uganda and South Africa aim to measure breast cancer and cervical cancer symptom and risk factor awareness and recognition and lay beliefs among urban and rural population in South African and Uganda. This is an important study, particularly in the context of the high breast and cervical burden in SSA where community awareness has not been assessed. However, I have concerns regarding the readability of the manuscript. The title does not incorporate what is being assessed in the study. It doesn’t specify that breast and cervical cancer risk factor and symptom awareness and lay beliefs are being measured and associated sociodemographic factors assessed.

Study outcomes are not well defined. There is limited description of how scores were organised and assessed for the analysis in the materials and methods, making it difficult to interpret results specifically for the multivariate analysis. Additionally, results are not well presented making it difficult to understand whether as a reviewer or as a reader. I am also concerned that not all results, most importantly multivariable results, are presented in tables included in the manuscript. The sample selection diagram to be revised and highlight/denote whether a household represents an individual. Additionally, the reported number of households assessed in text does not match with what is reported in the diagram.

The discussion needs to be considerably strengthened. Authors should try limiting repetition of results and focus more on discussing what the findings mean and relating it back to the aim of the study, how results compare to findings from previous studies or results from national programs and providing key recommendations.

There are several grammatical errors that the authors should correct. The authors should also cite appropriately in text and references to follow journal format.

An overall serious concern if the manuscript was to be considered for publication. There would be substantial revisions required. The authors need to be clear what the main aim of this study is and be concise about documenting study methods, reporting results and discussing the results.

**Abstract**

The tools should not be included in the background, it can be incorporated in the methods section as follows: “Data were collected by interviewers using electronic tablets customised with the the locally validated African Women Awareness of Cancer (AWCAN) tool.”

It would be helpful to mention test statistics used in your analysis.

Authors to report percentages up to one decimal point (i.e. 90.8% vs 91% for cervical cancer awareness)

I think findings serve as foundation for further studies and development of interventions.

**Background:**

line 73, “the” is missing, so the sentence should be “Breast and cervical cancer are the leading causes…”

Line 76-80, needs a reference

Line 80, Please write out in full before using abbreviations (UK and SA)

Line 80 - 82, This doesn’t read well. I understand that they present with late stage disease, but do you mean to convey that the main motivator for presenting for care are symptoms? if so please review this sentence so that this is communicated clearly.

Line 82-83, Please also include a reference for low survival rates for cervical cancer current reference included only relates to breast cancer survival rates.

Line 82 - 83, Perhaps “Late-stage at presentation is a major contributor to low survival rates” should be “Late-stage breast and cervical cancer at presentation is a major contributor to low survival rates”

Line 85 -86, This is not well-structured sentence. The independent clause should either come at the beginning or end of your compound complex sentence. I suggest you amend it as follows: “Early stage presentation is a key goal of comprehensive cancer policies as it enables more opportunities for curative treatment and improved prognosis a month those treated.”. Also, include a reference for this statement.

Line 86 -88, “Prompt help-seeking in the presence of symptoms…”. Help seeking should not only be motivated by symptoms. Prevention efforts should really be also addressing risk perception and perceived benefits for accessing screening.

Line 92, It should be “…nature of…” and “predictors” (plural). Additionally, it is simpler to just say “Understanding awareness of cancer symptom and risk factor is vital to underpin the development of locally relevant interventions”

Line 94, I assume “lay workers” should be “lay health workers”

Lin 95 -101, It will be useful to helpful to re current estimates present current breast/cancer awareness in Sub-Saharan Africa and specifically in the countries being studied (Uganda and South Africa). I know you mention that current estimates are mostly from hospital-based study populations, but even that can serve as starting point.

Line 103, I suggest changing “be very different” to “differ”

Line 104 -108, This should be part of Materials and method section. Instead, authors should put in the aim of the study here, before moving on to materials and methods.

**Materials and methods**

Line 113, “18 years and over” should be “18 years and older”

Line 117, There’s a comma missing after “AWACAN project”

Line 121, There’s a comma missing after “In this study”

Line 127-128, Please provide more details regarding the sampling approach including randomisations procedures. What if a household selected does not have a woman, or the woman fused? Also, what if the first woman approached has cancer history but there is another woman in the same household that doesn’t have a history of cancer?

Line 135 - 136, Were the interview done in private, as this can affect participant responses?

Line 140 – 141, I assume “education level” should be “highest education level”

Line 141, Employment status is called “paid work” in the tables – please use consistently in text and tables and ensure that it's defined accordingly in the text.

Line 143, Asset index should not be capitalised as it’s not a proper noun, please apply this consistently throughout text

Line 154 – 155, Please provide a brief provide description of questions asked to assess cancer awareness, similar to the approach used to describe knowledge of risk factors and symptoms in the subsequent sentence. Also, please provide a reference to the AWACAN breast and cervical cancer tool.

Line 157, “women’s” should be “woman’s” the “any” denotes singular, but women is plural

Line 158, “names” should be “name” it is a verb in this sentence

It would also be helpful to reference the relevant appendices for each item describe (i.e. breast cancer signs and symptoms (S3 appendix)

Line 168 – 172, More details are needed here regarding how the authors handled the scores and how they were interpreted, i.e. did higher scores indicate better/higher recognition of signs and symptoms? Also, how will these scores be reported and analysed, as continuous variable (averages/means/ medians) or categorize, (what cut-off are used)?

Line 178 – 179, was the consent form administered in the participants’ own/preferred language, and were consent forms translated?

Line 189, “Sociodemographic” does not have a hyphen which is inconsistent to previous format in the manuscript.

Line 191, The abbreviation IQR should come after ranges as it encompasses the word “range”

Line 192, up until now South Africa has been abbreviated to SA. Please choose format (abbreviated or written in full) and use consistently throughout the manuscript.

Line 195, “Sociodemographic” does not have a hyphen which is inconsistent to previous format in the manuscript.

Line 196 – 197, If I understand correctly, the authors dichotomised scores for awareness using the median as a cut-off for low and high based? Firstly, this should be described in detail in the “Measures of cancer awareness” section. Secondly, the authors should substantiate why they used this cut-off, and provide reference to support this, if any?

Line 194 -199 Logistic regression is not appropriate to use in this instance. Odd ratio has been shown to overestimate associations if the outcomes is not rare (i.e. above >=10% prevalence). I suggest using log binomial or Modified Poisson regression analysis to estimate relative risk. See below a reference that talks to this:

*Lovasi, G. S., Underhill, L. J., Jack, D., Richards, C., Weiss, C., & Rundle, A. (2012). At odds: concerns raised by using odds ratios for continuous or common dichotomous outcomes in research on physical activity and obesity. The open epidemiology journal, 5, 13.*

**Results**

S1 Appendix,

- The total household visited do not add up to 1941, they add up wot 1940 if you add up all the county totals
- Does a household represent an individual? This needs to be clarified in the methods and diagram.

Line 204, The overall refusal rate reported in text does not match what is reported in S1 Appendix

Line 205, Refusal rate for urban Uganda sites should be 13.7%

Line 209, Authors to include median age and IQR in Table 1.

Table 1,

- Categories for age should be labelled 18-29, 30-50, ≥50.
- Current Table 1 format is unnecessarily expansive. It should be adjusted to include number, with percentage in brackets in one column. (same applies to S1-S5 Appendices)
- It’s not necessary to include a total sample size for each variable. Instead. The sample size should only be included in the column titles for each country (i.e. total for each country, then totals for urban and rural area for each country).
- Information on missing data to be stated (if any).
- I find it odd that there no participants in a relationship but not living together, can the authors confirm this finding?

Line 211, Authors to report percentages up to one decimal point (i.e. 66.3% vs 66% for Paid work)

Line 221, Knowing of someone with the disease is not reported anywhere in the tables, and this was not defined in the materials and methods section.

Line 224, These results (9% of 1596) are not presented anywhere on the tables or appendices. There’s no total column in S3 appendix where I assume these results would be listed. The overall summary of these finding should also be included in one of the tables in the manuscript.

Line 224, These results (95%) are not presented anywhere on the tables or appendices. There’s no total column in S3 appendix where I assume these results would be listed. The overall summary of these finding should also be included in one of the tables in the manuscript.

Line 229 – 231, These results (overall commonly recognized and least recognized risk factors) are not presented anywhere on the tables or appendices. There’s no total column in S3 appendix where I assume these results would be listed.

Line 234, The figure included is not very clear. The risk factors are blurry and it’s difficult to read.

Line 240 – 243, These results (multivariable analyses) are not presented anywhere on the tables.

Line 248 -252, These results (overall breast cancer symptom and recognition) are not presented anywhere on the tables or appendices. There’s no total column in S3 appendix where I assume these results would be listed.

Line 255, “Education” should be “highest education level”. Employment should be “paid work”

Line 256 -259, These results (multivariable analyses) are not presented anywhere on the tables or appendices.

Line 260 -262, These results (multivariable analyses) are not presented anywhere on the tables or appendices.

Line 265, 20% should be21.0%

Line 267, 98% should be 97.8%

Line 268, include the following statement “…in prompted questions” before the bracket

Line 265, 1% should 0.8% and 22% should be 22.2%

Line 274, These results (38% of 1571) are not presented anywhere on the tables or appendices. There’s no total column in S5 appendix where I assume these results would be listed.

Line 276, Having unprotected sex is the most commonly recalled risk factor in urban SA. In Rural SA, it is having other sexually transmitted diseases.

Line 277 - 278, These results (<1%) are not presented anywhere on the tables or appendices. There’s no total column in S5 appendix where I assume these results would be listed.

Line 281 - 284, These results are not presented anywhere on the tables or appendices. There’s no total column in S5 appendix where I assume these results would be listed.

Line 286 – 287, 8% should be 7.9%, 27% should be 26.6%, 38% should be 37.8%, and 31% should be 30.7%

Line 289, The figure included is not very clear. The risk factors are blurry and it’s difficult to read.

Line 292 – 294, These results (multivariable analyses) are not presented anywhere on the tables.

Line 296 (page 18), These results (multivariable analyses) are not presented anywhere on the tables.

Line 297, These results are not presented anywhere on the tables or appendices. There’s no total column in S5 appendix where I assume these results would be listed.

Line 301, The figure included is not very clear. The risk factors are blurry and it’s difficult to read.

Line 304 – 310, These results (multivariable analyses) are not presented anywhere on the tables.

Line 314, 93% should be 92.3%, and 34% should be 33.7%,

Line 316, 85% should be 85.4%

Line 317 – 318, <1% should be overall <0.5%, and 22% should be 22.3%

**Discussions**

Line 328, “In keeping with other studies…) should be “In line with findings from previous studies, )

Line 329, “…symptom awareness was higher with prompted questioning”

Line 332, This paragraph should be summarised in the limitations.

Line 344 - , should move to line 328, after the first paragraph of the discussion

Line 347, better to say responsive rather than amenable. Also, can you include reference that support the statement regarding exercise as an effective intervention for being overweight.

Line 348, The authors should include reference for breast cancer risk factors being common to other NCDs. Also, it is generally more cost effective to adapted existing interventions to include additional messaging. The authors should maybe highlight this seeing as there are risk factors common to other NCDs.

Line 355, There should be a common after the reference not a full stop.

Line 362 - 364, References after need here, including the respective guidelines.

Line 367 – 368, References needed for the statement that screening coverage is suboptimal is SSA.

Line 369, There should not be a comma after screening. Also, authors should include the SA cancer prevention guidelines as a reference here in addition to the current reference.

Line 373, What do the authors mean by opportunistic screening programs? This needs to be clarified, and the reasons why it seems to mostly be limited to national and regional referral hospitals. Remove the bracket after 7%. Also, 7% should be 7.0%

Line 375, A reference is needed for program screening HIV infected women more frequently.

Line 376, I would rather say findings are unexpected, not disappointing. How do these results compare to previous results, or reported results from national programs? Also, Authors should explore the reasons why HIV was not well recognised as a risk factor for HIV among the study population. Is there data on HIV prevalence amongst your study participants? If not, I’m sure that there are HIV estimates for communities involved in the study? Seeing as SSA has highest burden of HIV, one would expect that exposure to HIV service would lead to some exposure to cervical cancer prevention messaging seeing as HIV infected women are at higher risk. Additionally, cervical cancer prevention and screening guidelines are integrated into HIV treatment guidelines in SA (since 2010), as should screening and treatment services for HIV infected women. Granted access to integrated services may be limited in rural areas, but it still doesn’t explain the low recognition of HIV as a risk factor for cervical cancer.

Line 383, Authors to include references for SA and Uganda HPV vaccine programs.

Line 381 – 389, Again, authors need to discuss how these results compare to previous studies or reported results from national programs.

Line 391 – 397, Again, authors need to discuss how these results compare to previous studies or reported results from national programs. The Gini coefficient explains the unequal distribution of wealth between communities, but more needs to be said regarding how this may lead to unequal health care service access, including prevention messaging and screening services which may differ in these circumstances.

Line 413 – 415, Additionally, witchcraft related beliefs are community specific, and this also need to be considered in future research related to this topic.

Line 416 – 419, There’s been previous studies related to insertion of objects for example in HIV prevention studies (related to condom use/non-use). Though this doesn’t relate to cervical cancer directly, it may provide some information on issues related to health risk behaviour among people that believe in such practices which authors may compare and contrast to findings in the current study.

Line 424 – 438, This needs to be more concise, there’re repetitions.

Line 440 – 446, Include the issue regarding prompted vs. unprompted questioning as a limitation.
